# Supplementary material for: Quantifying negative selection on synonymous variants
Source: HGG Adv. 2024 Jan 8;5(2):100262. doi: 10.1016/j.xhgg.2024.100262 (PMC10835449; doi:10.1016/j.xhgg.2024.100262)
Supplement: Document S1. Figures S1–S12, Tables S1 and S2, and Notes S1 and S2 [file mmc1.pdf]

HGGA, Volume *5*

Supplemental information

Quantifying negative selection on synonymous variants

Mikhail Gudkov, Loïc Thibaut, and Eleni Giannoulatou

Supplemental Figures

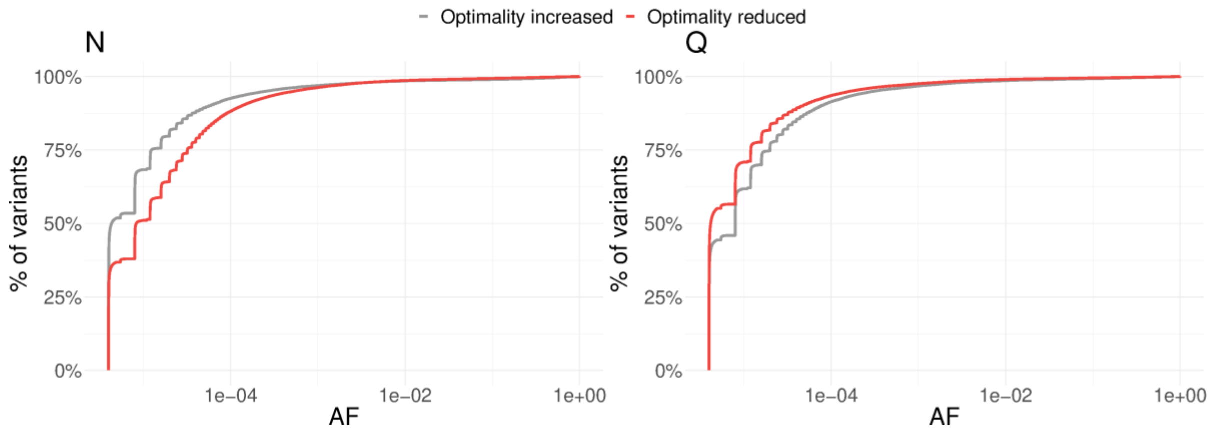

Figure S1. Example of a difference in allele frequencies of variants due to different mutability. Compared to glutamine (Q), the proportion of singletons in asparagine's (N) optimality-reducing variants (red line) is relatively low because of the contribution of the highly mutable CpGs transitions ("xxC->xxT").

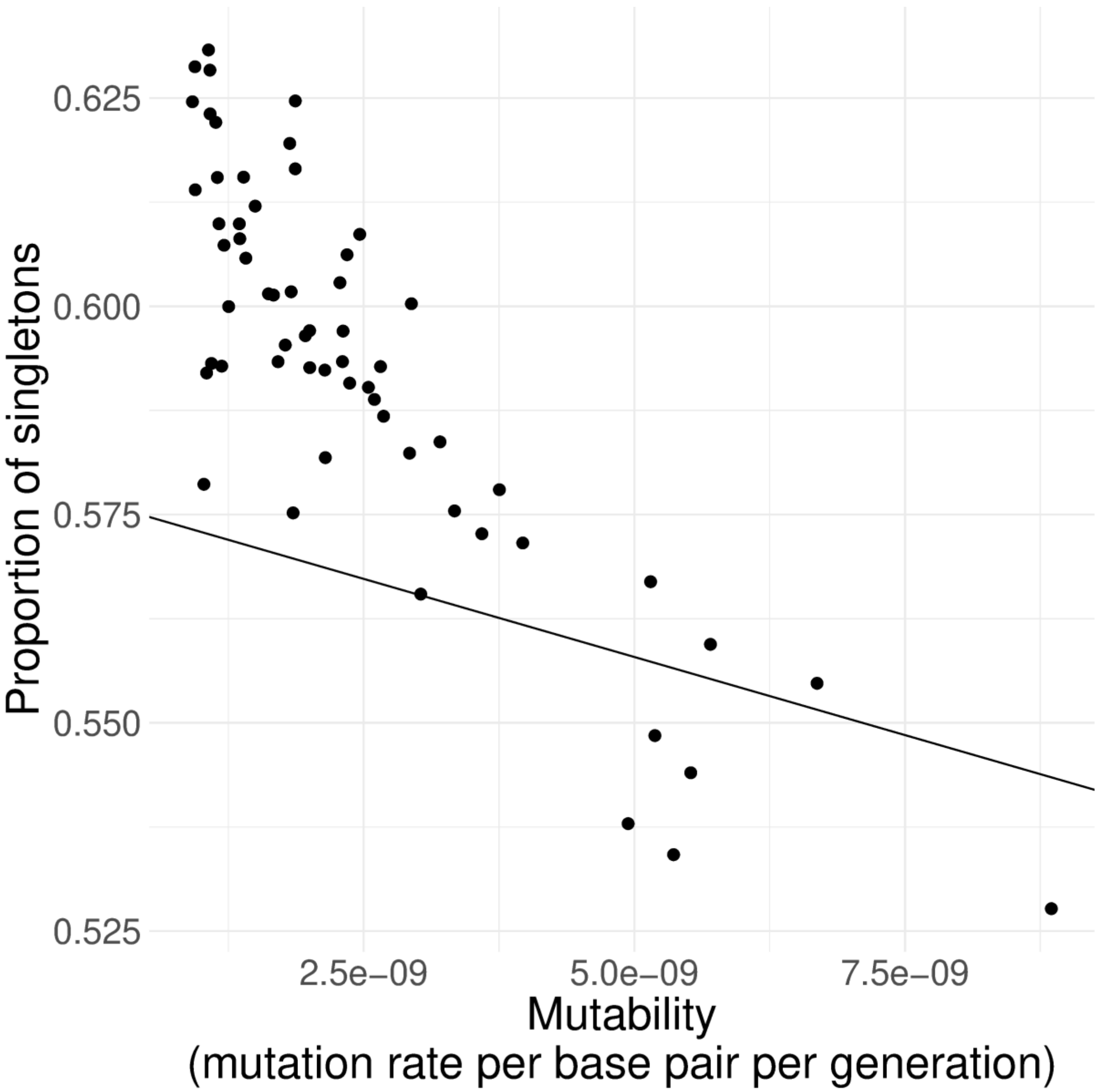

Figure S2. Bias towards synonymous transversion variants in the MAPS model. The line indicates the slope of the MAPS model calibrated on all synonymous variants (transversions, CpGs and non-CpG transitions). Each dot corresponds to a unique mutability value that a transversion variant can take.

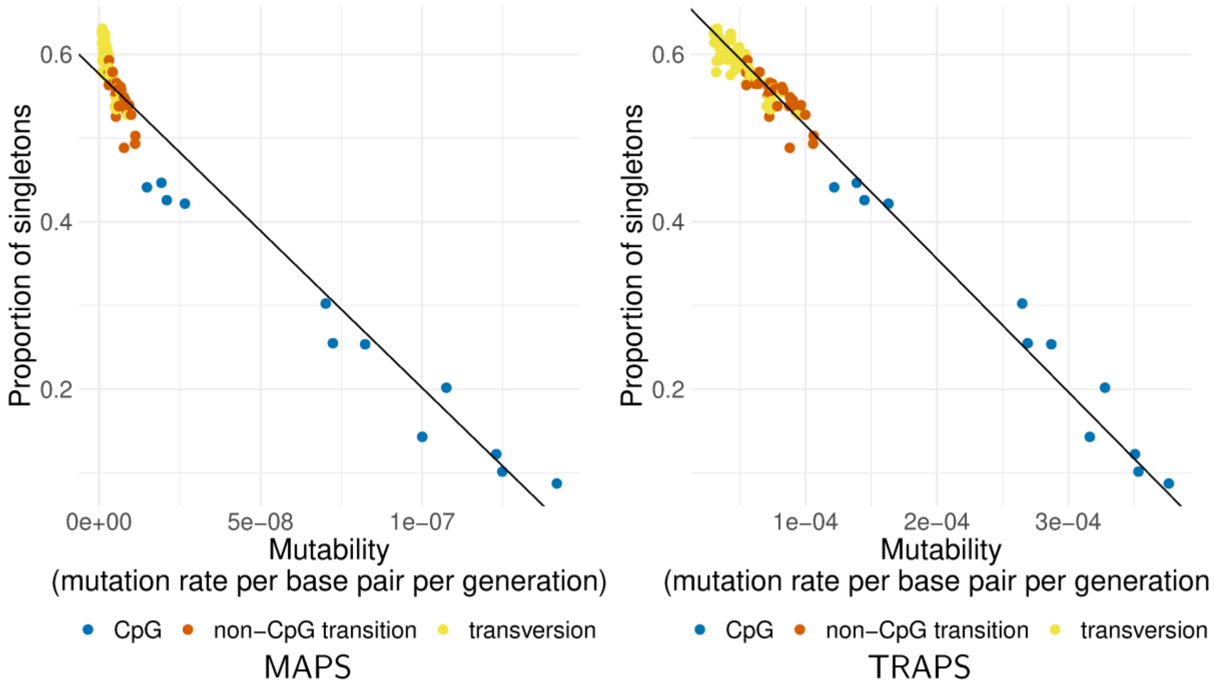

Figure S3. The effect of square-root transformation on the fit of the MAPS model of the expected number of singletons.

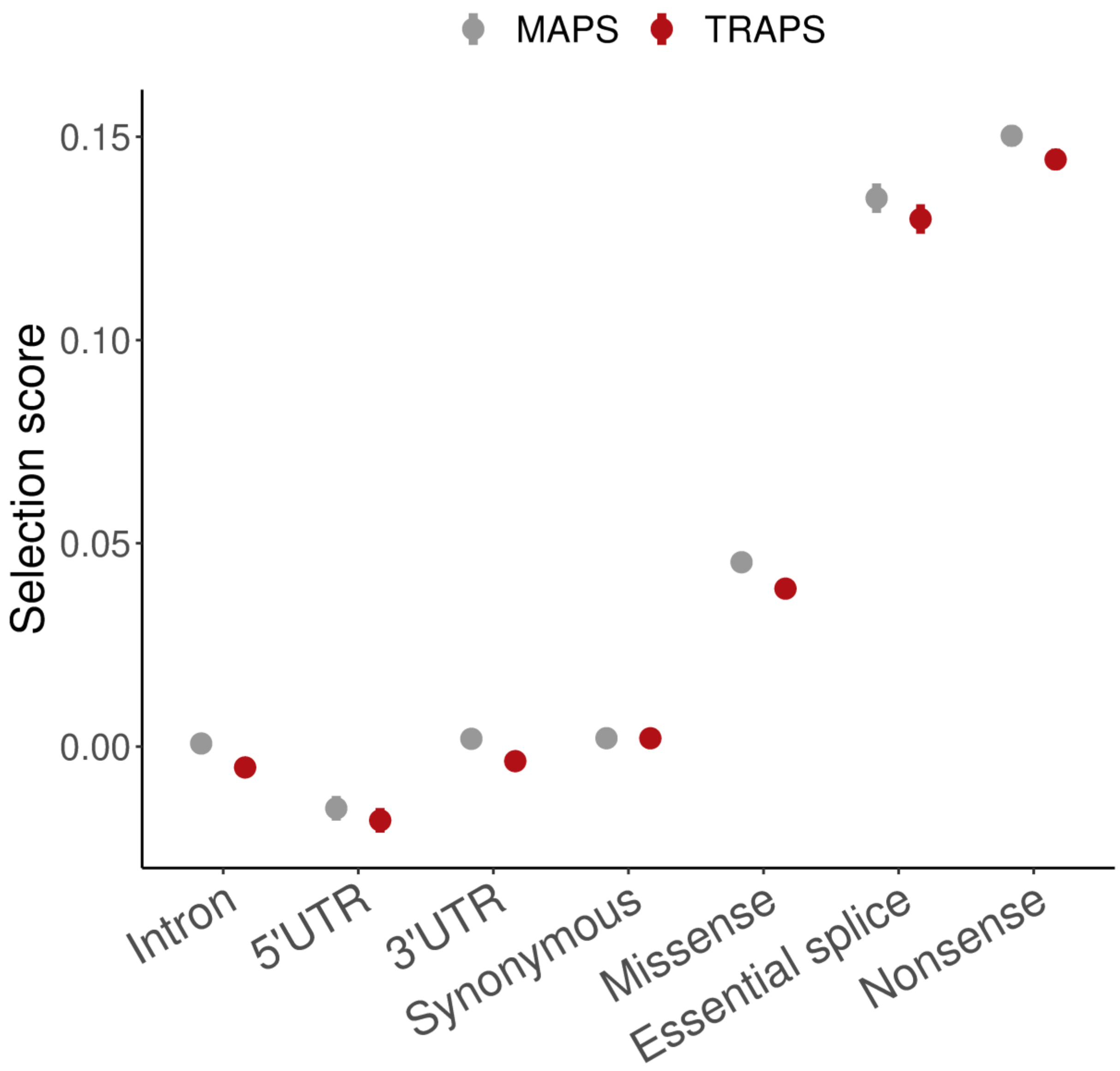

Figure S4. Estimates of negative selection in SNVs by variant class, calculated using MAPS and TRAPS. Error bars are 95% binomial confidence intervals.

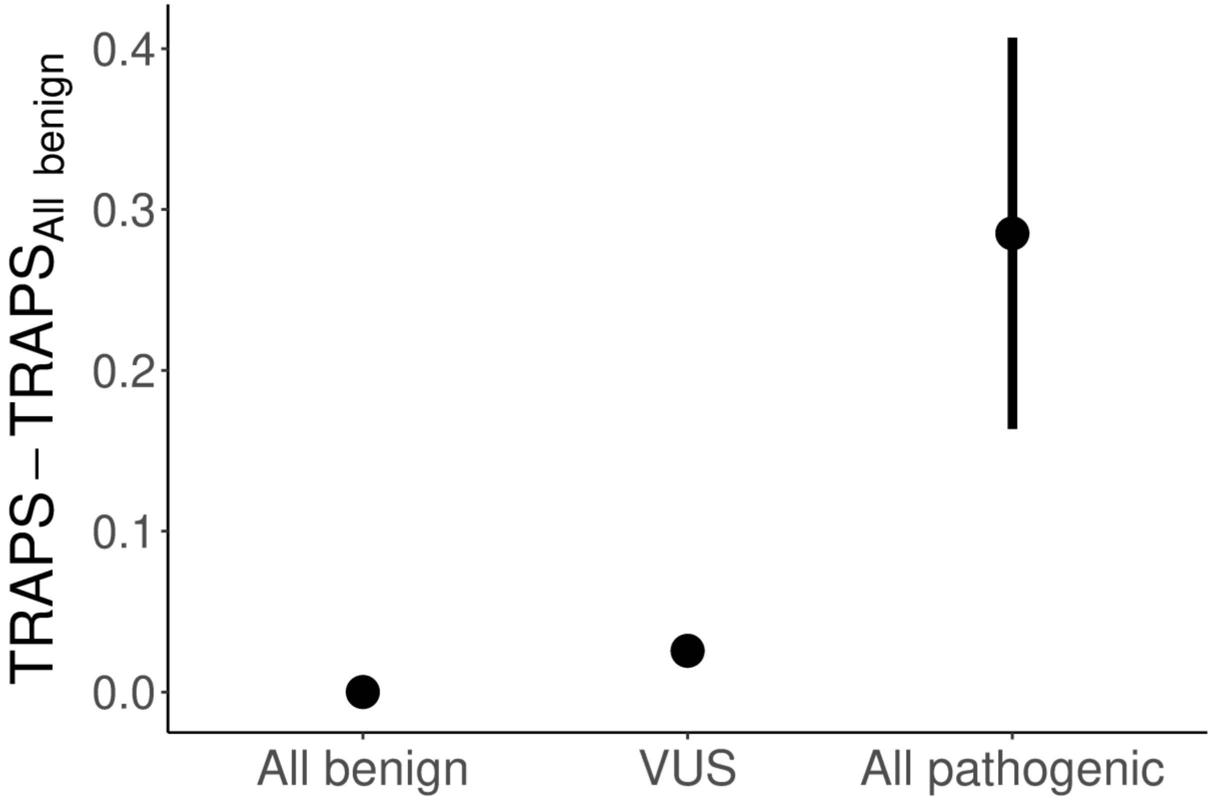

Figure S5. *Δ*TRAPS scores for synonymous variants from ClinVar (version "2019-07") by category, with "All benign" variants used as a reference. Error bars are 95% binomial confidence intervals.

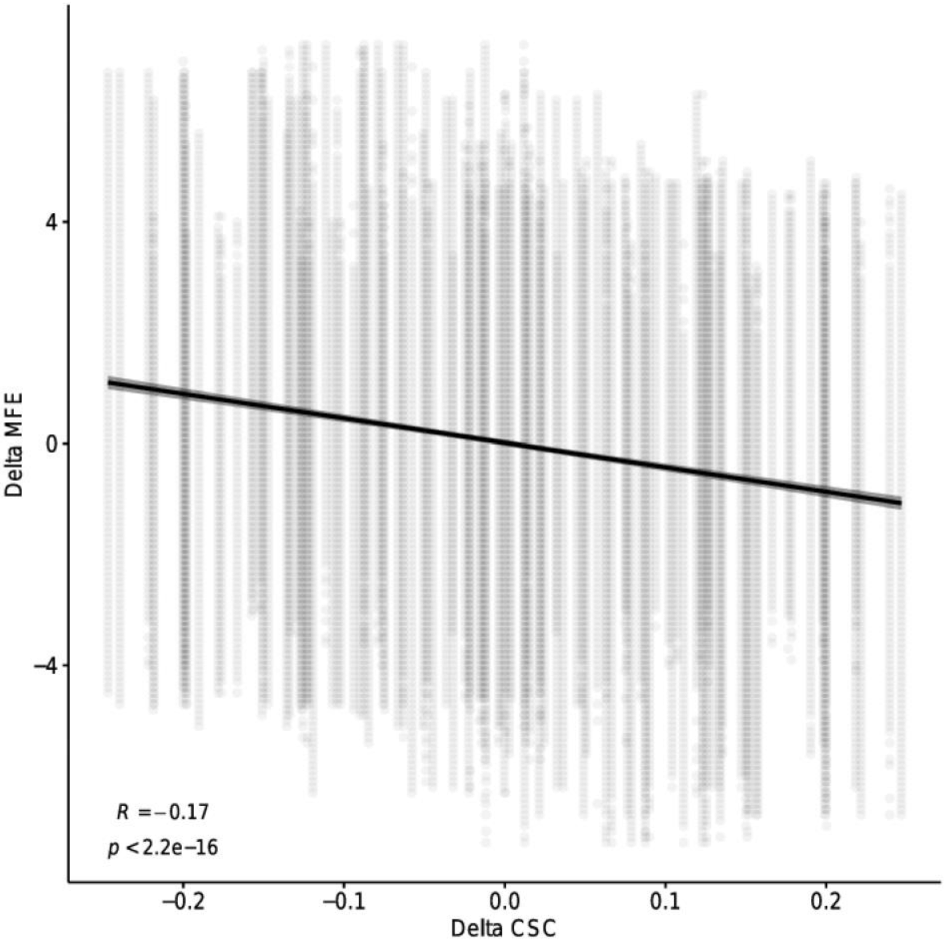

Figure S6. Correlation between ΔCSC (Codon Stability Coefficient) and ΔMFE (Minimum Free Energy), showing that positive changes in CSC (stabilising variants) tend to correspond to negative changes in Minimum Free Energy.

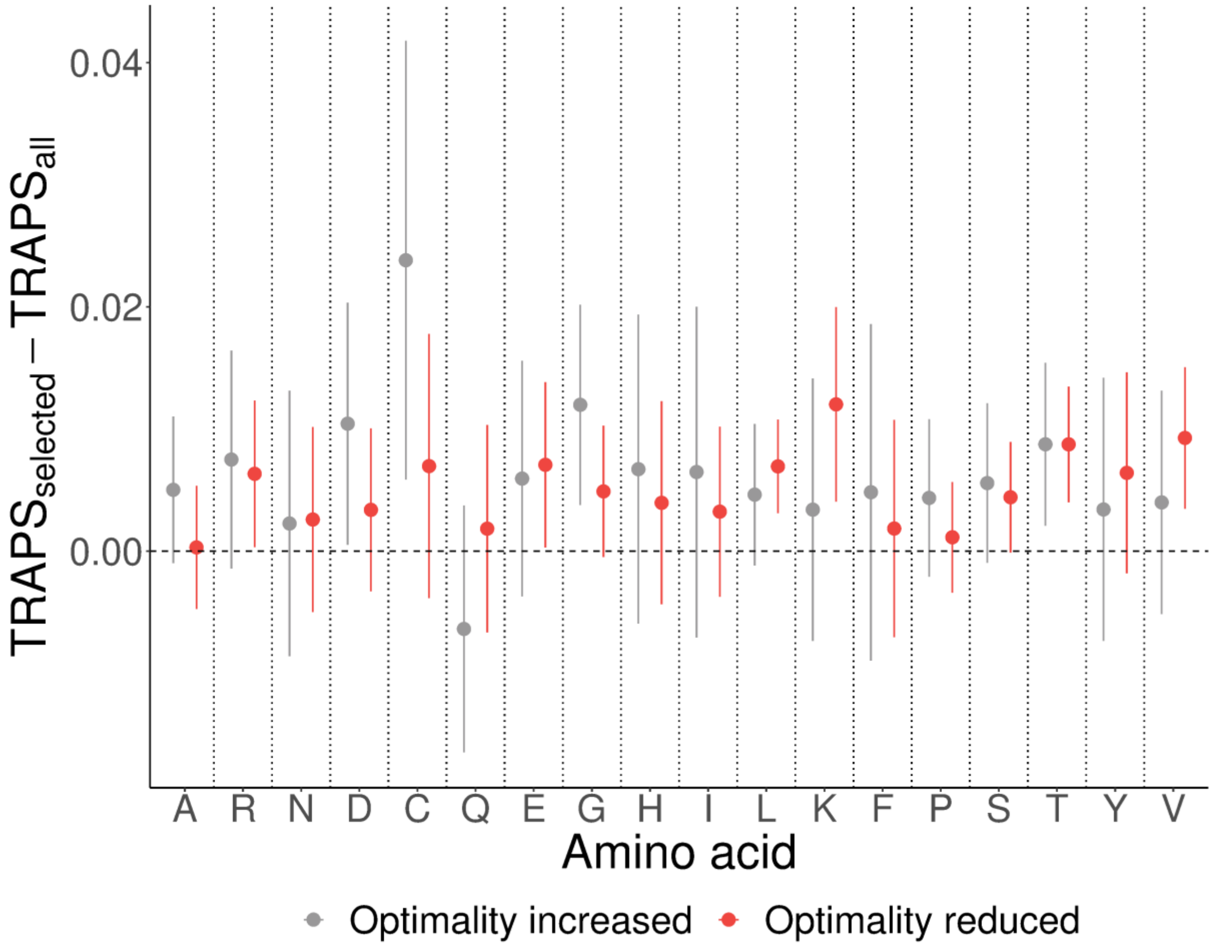

Figure S7. Difference between TRAPS scores in 30% most constrained genes (based on the LOEUF metric of intolerance) and in all genes. Error bars are 95% binomial confidence intervals.

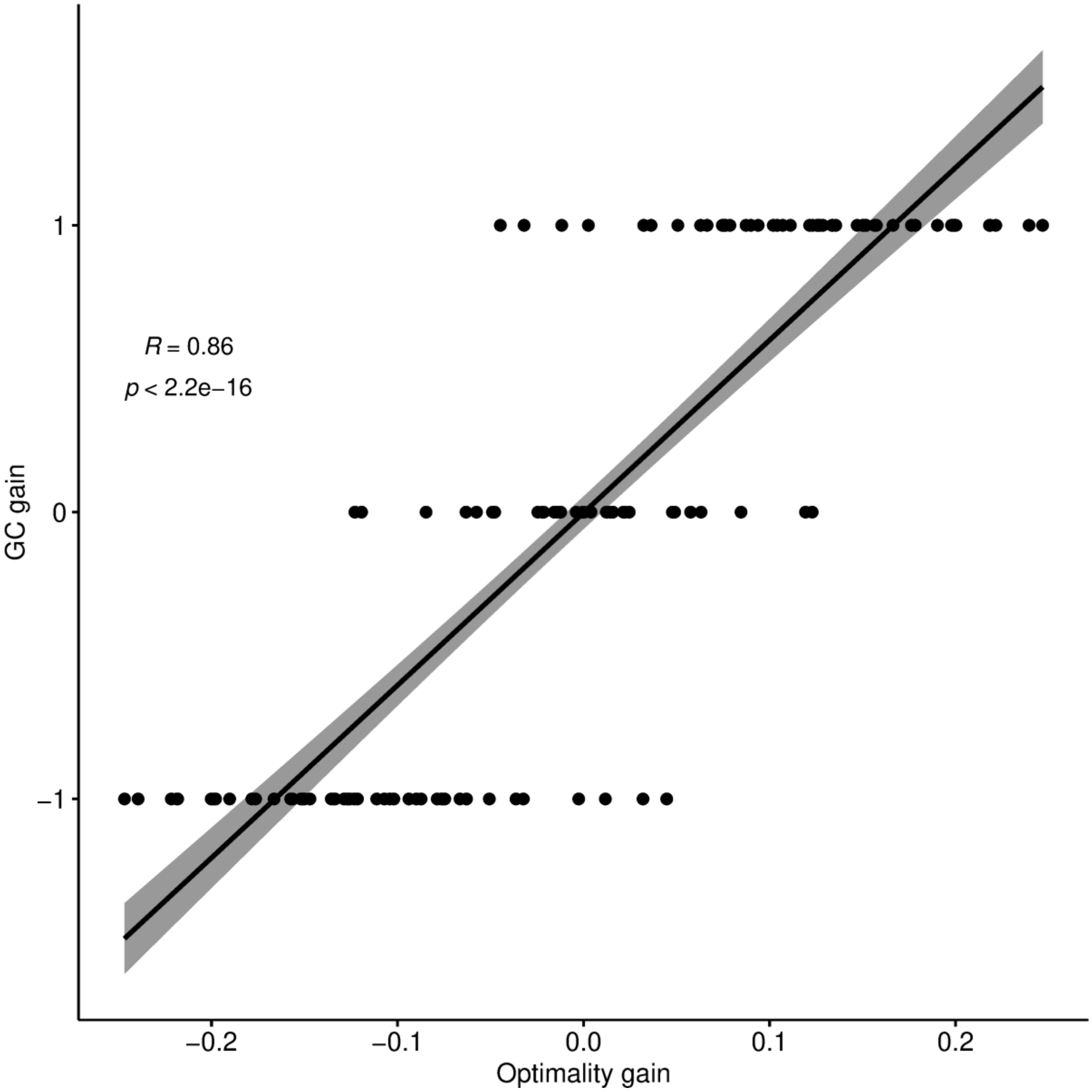

Figure S8. Correlation between optimality gain (*Δ*CSC) and change in GC content. A GC change of "-1" ("+1") indicates a G/C to A/T (A/T to G/C) substitution.

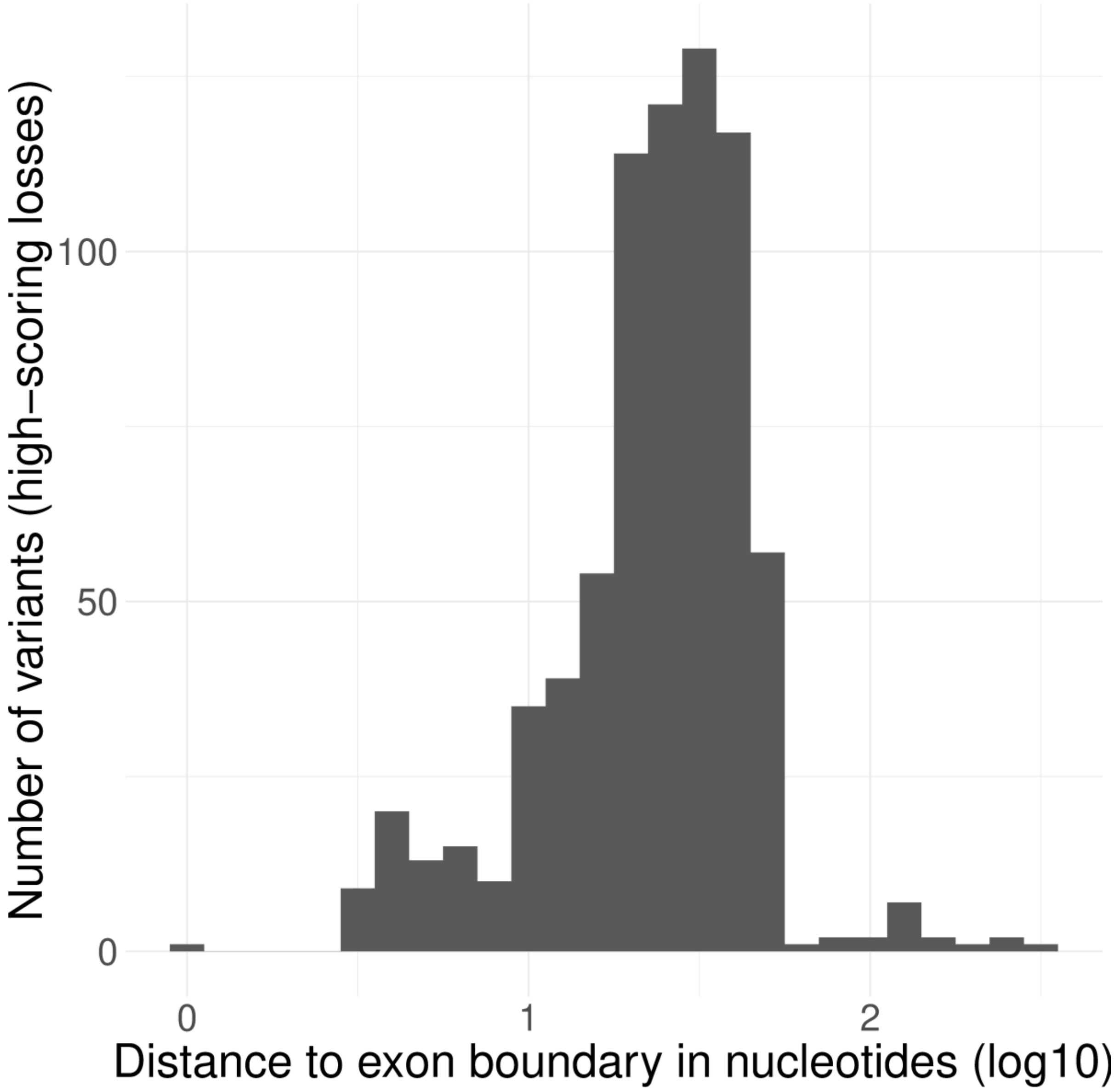

Figure S9. Distance to nearest exon junction boundary in high-scoring synonymous splice-site losses. Values are shown on logarithmic scale.

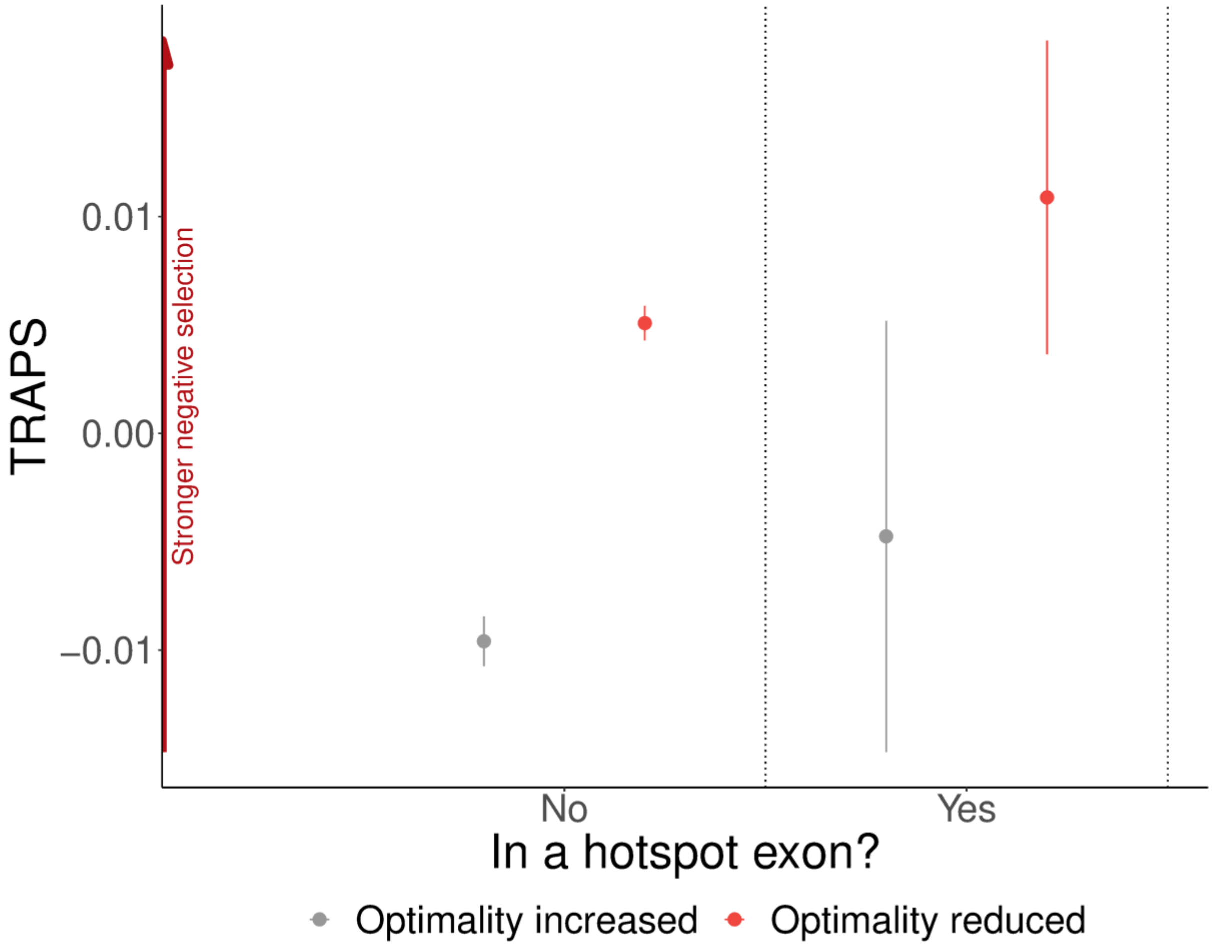

Figure S10. TRAPS scores for optimality-reducing and optimality-increasing synonymous SNVs within and outside of splicing hotspot exons.

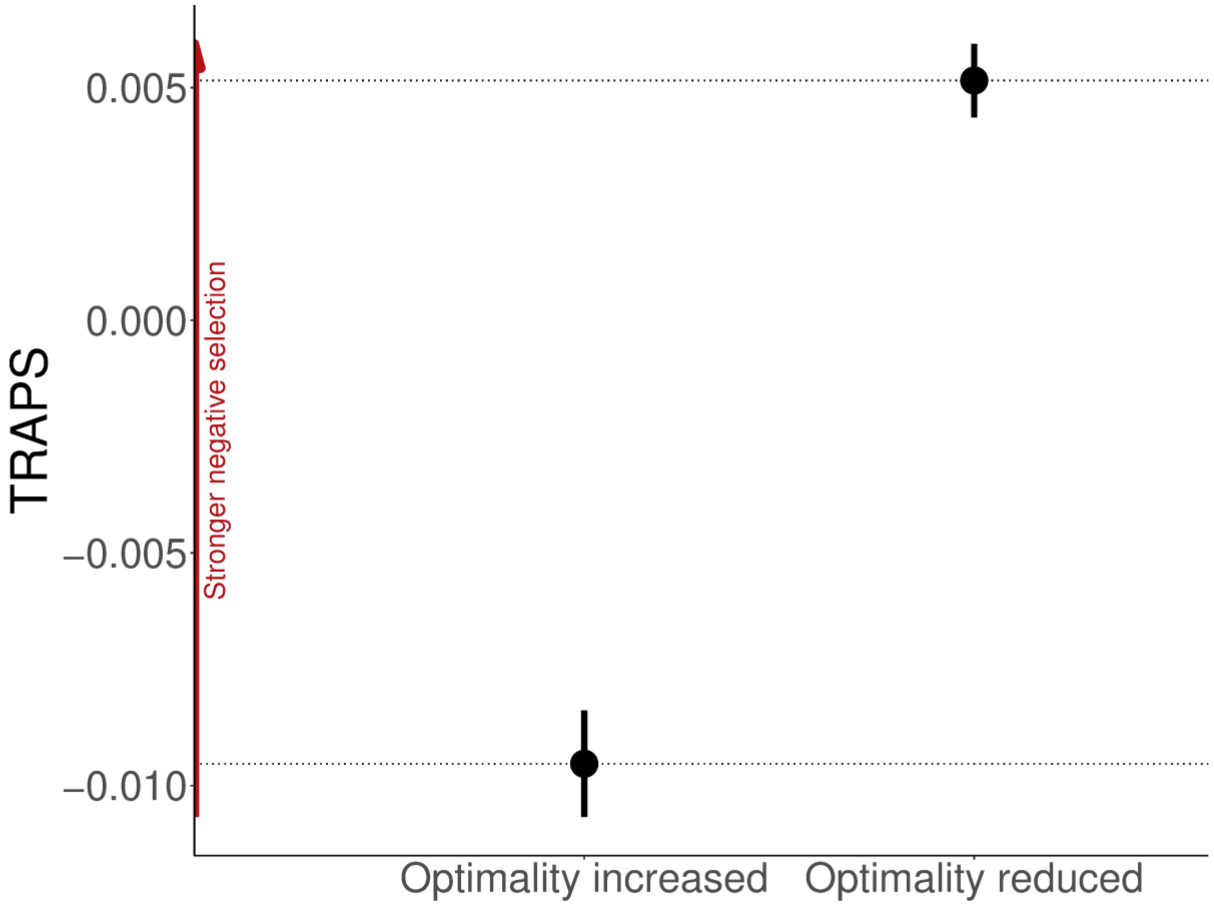

Figure S11. Averaged TRAPS scores of all optimality-reducing and optimality-increasing synonymous SNVs. Error bars are 95% binomial confidence intervals.

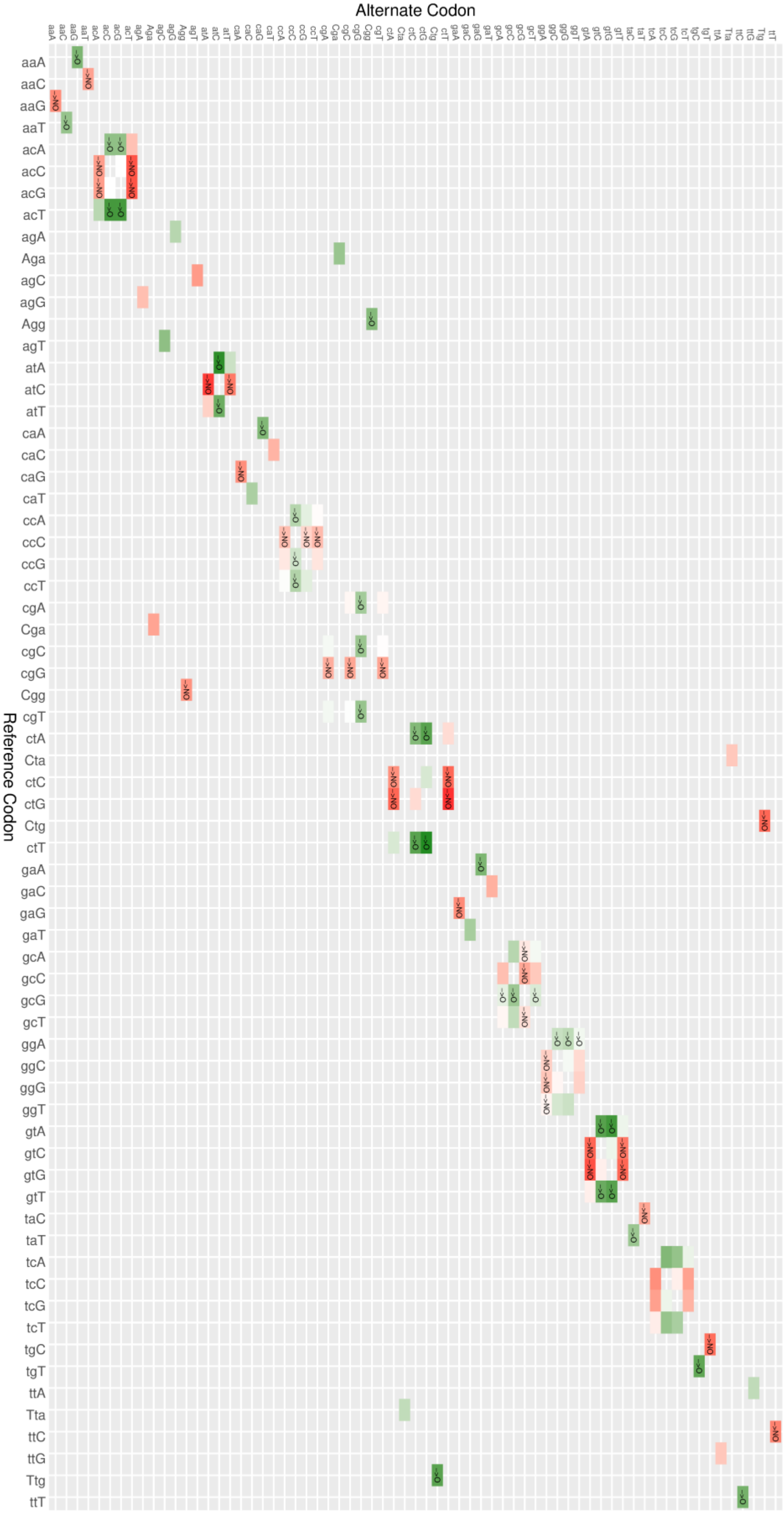
Alternate Codon
Reference Codon
aaA
aaC
aaG
aaT
acA
acC
acG
acT
agA
Aga
agC
agG
Agg
agT
atA
atC
atT
caA
caC
caG
caT
ccA
ccC
ccG
ccT
cgA
Cga
cgC
cgG
Cgg
cgT
ctA
Cta
ctC
ctG
Ctg
ctT
gaA
gaC
gaG
gaT
gcA
gcC
gcG
gcT
ggA
ggC
ggG
ggT
gtA
gtC
gtG
gtT
taC
taT
tcA
tcC
tcG
tcT
tgC
tgT
ttA
Tta
ttC
ttG
Ttg
ttT

Figure S12. Yield in optimality for all codon transitions based on CSC in HEK293T cells. Transitions that produce a more optimal codon are coloured green, whereas optimality-reducing transitions are coloured red. When the produced and alternate codons have different signs, such transition is marked with either “->O” (optimality-increasing) or “->NO” (optimality-reducing).

Supplemental Tables

Table S1. Difference in GERP distributions between optimality-reducing and optimality-increasing synonymous variants in two-codon amino acids. Bonferroni-adjusted p-values shown are from one-sided Mann-Whitney-Wilcoxon test. All genes.

Amino acid	Difference in medians	Wilcoxon p-value
Q	3.6	<0.0001
Y	1.9	<0.0001
H	1.8	<0.0001
K	1.4	<0.0001
N	1.3	<0.0001
E	1.1	<0.0001
F	0.92	<0.0001
D	0.88	<0.0001
C	0.51	<0.0001

Table S2. Predictors of the negative selection captured by TRAPS.

Predictor	Variance explained (%)	Selected by LASSO
tAI gain	16.48	Yes
CSC gain	4.22	Yes
GC gain	0.74	Yes
Mutability	–	No
Number of codons	–	No

Supplemental Note 1: gnomAD and TRAPS

We applied several criteria in the filtering and preprocessing of gnomAD data. We only retained those synonymous variants that were compliant with quality control (non-zero allele frequency and “PASS” filter flag) and had “synonymous_variant” as their most severe transcript consequence. The latter condition removes all pLoF SNVs, including variants in canonical splice sites. We additionally checked that all those variants have a protein-coding transcript where they are annotated as synonymous.

We also applied an allele number threshold to make sure that we only include those SNVs that were called in at least 80% of potential carriers. For example, for chromosome Y variants, we excluded all variants where the call was made in less than 80% of the total of 67,961 potential

male carriers, whereas for variants in autosomes, the number was 80% of two times the total number of male and female samples (125,748).

We used the same methylation scale as in the 2020 gnomAD flagship paper with values 0, 1 and 2 to distinguish between unmethylated regions and regions with medium or high levels of methylation.

Supplemental Note 2: LASSO

We used the least absolute shrinkage and selection operator (LASSO) method to perform variable selection in a linear model with negative selection (TRAPS) scores as the predictor variable and several potential predictors of this selection as explanatory variables.

We first precomputed the optimal value for the regularisation parameter λ minimising the mean-squared error using 10-fold cross-validation. Using the obtained value, we then applied LASSO, which removed such variables as the total number of codons and mutability, while keeping tAI, CSC and GC gain variables (Table S2).
