# Supplementary material for: Quantifying negative selection on synonymous variants
Source: HGG Adv. 2024 Jan 8;5(2):100262. doi: 10.1016/j.xhgg.2024.100262 (PMC10835449; doi:10.1016/j.xhgg.2024.100262)
Supplement: Document S2. Article plus supplemental information [file mmc2.pdf]

HGG Advances ARTICLE

Quantifying negative selection on synonymous variants

Mikhail Gudkov,[1,2] Loïc Thibaut,[1,3] and Eleni Giannoulatou[1,2,4,*]

Summary

Widespread adoption of DNA sequencing has resulted in large numbers of genetic variants, whose contribution to disease is not easily determined. Although many types of variation are known to disrupt cellular processes in predictable ways, for some categories of variants, the effects may not be directly detectable. A particular example is synonymous variants, that is, those single-nucleotide variants that create a codon substitution, such that the produced amino acid sequence is unaffected. Contrary to the original theory suggesting that synonymous variants are benign, there is a growing volume of research showing that, despite their "silent" mechanism of action, some synonymous variation may be deleterious. Here, we studied the extent of the negative selective pressure acting on different classes of synonymous variants by analyzing the relative enrichment of synonymous singleton variants in the human exomes provided by gnomAD. Using a modification of the mutability-adjusted proportion of singletons (MAPS) metric as a measure of purifying selection, we found that some classes of synonymous variants are subject to stronger negative selection than others. For instance, variants that reduce codon optimality undergo stronger selection than optimality-increasing variants. Besides, selection affects synonymous variants implicated in splice-site-loss or splice-site-gain events. To understand what drives this negative selection, we tested a number of predictors in the aim to explain the variability in the selection scores. Our findings provide insights into the effects of synonymous variants at the population level, highlighting the specifics of the role that these variants play in health and disease.

Introduction

Despite the ongoing efforts in studying the relationship between genetic variants and their phenotypic manifestations, to date there remains a substantial portion of developmental abnormalities that cannot be attributed to a specific genetic cause. This can be partially explained by the fact that for every unsolved disease, there can be myriads of candidate variants with an unknown potential to contribute to the development of detrimental phenotypes.

In turn, genetic variant prioritization is complicated by the need to assess single-nucleotide variants (SNVs) based on their possible effects. Even though this process may be straightforward for predicted loss-of-function (pLoF) variants, such as stop-gain variants, as well as for SNVs with known and experimentally validated effects on protein structure and function, there are some classes of genetic variation that tend to affect cellular processes by means that are, as of yet, understudied.

In particular, synonymous genetic variants—that is, those SNVs that replace a codon with a different one encoding the same amino acid—are routinely considered to be nondeleterious. However, it is well established that synonymous codons undergo selection in the human genome,[1,2] and therefore a substitution of one codon for another at any position is likely to produce significant effects.

Apart from their impact on the splicing[3–6] and micro-RNA-binding processes,[7] synonymous variants can alter the structure (and thus the stability) of the produced mRNA transcript.[8,9] Moreover, synonymous variants are known to affect the efficiency of translation and to modify the properties of the nascent protein, such as its structural conformations influenced by co-translational folding and phosphorylation status.[10–13] These translation-induced modifications to the properties of the produced protein are particularly prominent in the context of enzymes.[14]

Incidentally, it is possible that synonymous SNVs are not completely "silent," as it has been shown that synonymous codons can leave a unique footprint in the form of backbone torsion angles in the structure of the encoded amino acid.[15] Hence, synonymous variants are potentially traceable even beyond the DNA sequence level.

Since synonymous variants manipulate the natural codon usage signatures of DNA sequences, it is important to characterize those codon substitutions. Frequency-based metrics, like the codon adaptation index (CAI[16]) and relative synonymous codon usage (RSCU)[17] provide a convenient way of working with synonymous codons based on how often they appear in genetic sequences. However, those metrics do not explicitly account for functional importance. In contrast, metrics like the tRNA adaptivity index (tAI[18,19]) and codon stability coefficient (CSC[20]) each focus on different processes that synonymous codons are involved in and provide the basis for

[1]Victor Chang Cardiac Research Institute, Darlinghurst, NSW 2010, Australia; [2]St Vincent's Clinical School, UNSW Sydney, Sydney, NSW 2052, Australia; [3]School of Mathematics and Statistics, UNSW Sydney, Sydney, NSW 2052, Australia
[4]Lead contact
*Correspondence: e.giannoulatou@victorchang.edu.au
https://doi.org/10.1016/j.xhgg.2024.100262.
© 2024 The Authors. This is an open access article under the CC BY license (http://creativecommons.org/licenses/by/4.0/).

grouping codons based on their functional optimality. Specifically, the tAI provides a measure of relative availability of tRNA molecules for each codon, whereas CSC estimates the level of correlation between the usage of a codon in a transcript and the experimentally derived stability measurement for that transcript. It is, therefore, possible to assess what effect a synonymous variant may have on translational efficiency and mRNA stability, based on tAI- and CSC-defined codon optimality, respectively.

It has been shown that synonymous variants that reduce codon optimality undergo purifying selection.[21–23] However, the factors influencing this selective pressure remain insufficiently investigated. This knowledge gap presents a significant limitation to uncovering the functional relevance and impact of synonymous variants. To fully understand the potential role of synonymous variation in human disease, a quantitative population-level framework is needed to assess what shapes their deleteriousness in comparison to other, better-studied classes of SNVs.

In this work, we quantified the intensity of the negative selection acting on synonymous variants using a modified version of the mutability-adjusted proportion of singletons (MAPS) metric, which we called TRAPS (transformed, adjusted proportion of singletons). Our variant set comprised all high-confidence synonymous SNVs reported in the gnomAD v.2 database.

We found that purifying selection operates on synonymous variants in an effect- and amino-acid-dependent manner, which was confirmed using conservation analysis. We also show that this selection can be partially explained using a set of predictors. According to our model, some of the key predictors of the negative selection on synonymous variants include the variant's effect on mRNA stability and the reduction in the tRNA availability for the mutated codon, as well as the induced change in local GC content. Furthermore, we showed that synonymous variants with the potential to create cryptic splice-site losses or splice-site gains are subject to strong selection.

Material and methods

Quantifying codon optimality

Because of the redundancy in the genetic code, most amino acids are encoded by more than one codon, with some codons being more optimal than others. CSC is a popular measure of codon optimality calculated as the Pearson correlation between each codon's frequency in a transcript and the resultant stability of the transcript, defined in terms of its half-life time.[20] When CSC is used, a codon is considered optimal if its CSC is positive. This definition of optimality assumes that codon-optimized transcripts have increased mRNA stability than less-optimized transcripts. In this work, we used CSC scores pre-calculated for human embryonic kidney 293T (HEK293T) cells.[24]

Due to the ambiguity of the definition of a nonoptimal codon in the case of the histidine amino acid, where both of the two codons have negative CSC scores, we opted to classify synonymous variants as either optimality reducing (i.e., creating a codon with lower CSC) or optimality increasing (i.e., creating a codon with higher CSC) instead of using the "optimal-to-nonoptimal"/ "nonoptimal-to-optimal" dichotomy.[21]

Apart from mRNA stability, synonymous variants can have an effect on the speed of translation by creating so-called "translational pause sites" caused by low tRNA availability.[17] Indeed, it has been shown that selective pressure has optimized the positions of some codons to better match local availability of tRNAs to reduce the consequences of translational errors.[25] One useful metric of tRNA availability is the tAI.[18,19] The tAI of a codon is the estimated availability of the tRNAs that participate in translating that codon. Given the high level of correlation between tRNA expression and the number of copies of the corresponding tRNA gene, tRNA abundance is often approximated using tRNA gene copy number. Using this definition of optimality, more optimal codons correspond to more abundant tRNAs.

To aid in the analysis of optimality-altering variants, we also downloaded a dataset of genome-wide codon usage, namely CoCoPUTs v.1.2,[26] and used it to calculate the average codon usage bias in the genome for all amino acids.

Quantifying purifying selection

MAPS is a metric of deleteriousness that has been extensively used for quantitatively predicting the effects of missense and pLoF variants.[27,28] MAPS was developed as an improvement of the originally proposed proportion singleton metric, which was known to be biased toward non-CpG variants. MAPS scores are calculated as the scaled excess or deficit of singletons (variants with allele count of 1), where the expected number of singletons is taken from a linear model, calibrated on a group of supposedly more neutral variants (e.g., synonymous), and weighted by the total number of observations in each mutational context. The model assumes that if a particular variant is deleterious, then it will be rare in the population because selective pressure will decrease its frequency in the population over time. MAPS is designed in such a way that higher values indicate an enrichment of singletons, which suggests higher constraint.

The latest version of the MAPS model uses methylation and mutability adjustment to estimate how likely any particular SNV is to occur and eliminate the bias toward the more mutable CpG variants.[27,29] Indeed, CpGs are known to have mutation rates about 100 times higher than non-CpG transitions and transversions (changes from a purine base to a pyrimidine base, or vice versa). Intuitively, as CpGs have higher chances to mutate acceptably, it is expected that this class of variants should have fewer singletons.

The use of the relative number of singletons as a proxy for variant's pathogenicity in MAPS is justified by the fact that human populations are protected against pathogenic variants by negative selection, which is constantly pushing these variants to the lower bound of the allele frequency spectrum. Even though based on the assumptions of the infinite sites model the number of singletons should not vary with mutability, this model is not applicable in the context of the gnomAD database due to the increasing saturation of all possible synonymous variants and mutational recurrence.[30] Figure S1 illustrates this phenomenon.

We fit the MAPS model on a subset of over 2 million synonymous SNVs from 125,748 gnomAD exome sequences (release 2.1.1). Using annotations from the Ensembl Variant Effect Predictor,[31] we retained only those variants that had "synonymous variant" as their most severe consequence, thus removing all

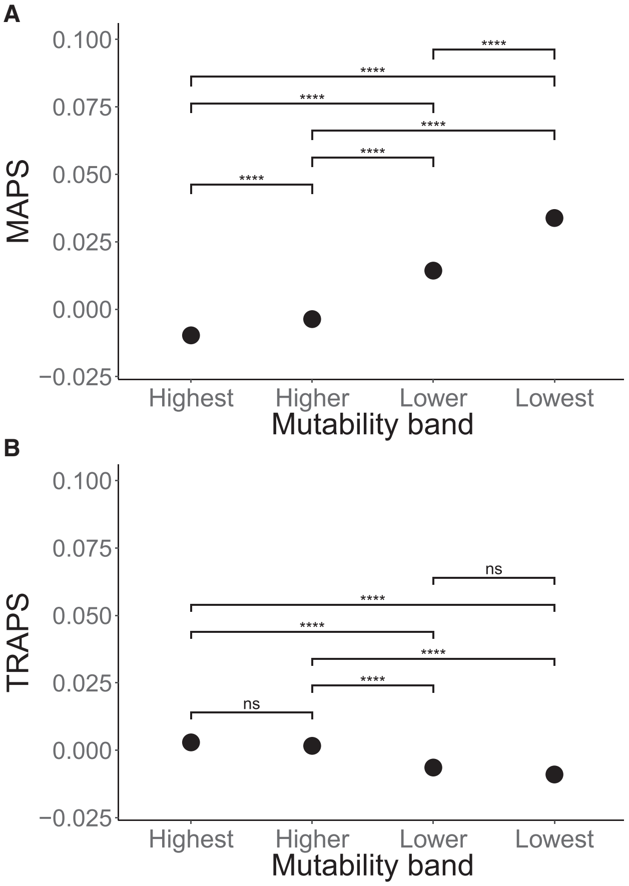

Figure 1. Comparison of the performance of mutability correction in MAPS and TRAPS
Averaged MAPS (A) and TRAPS (B) scores are shown for each mutability quartile: "lowest" (0%–25%, transversions), "lower" (25%–50%, mix of non-CpG transitions and transversions), "higher" (50%–75%, mix of non-CpG transitions and transversions), and "highest" (75%–100%, CpG and non-CpG transitions and transversions). Bonferroni-adjusted p values are shown for each comparison (Welch modified two-sample t test): *p $<$ 0.05, **p $<$ 0.01, ***p $<$ 0.001, ****p $<$ 0.0001, and ns, not significant.

missense and pLoF SNVs, including those implicated in the disruption of canonical splice sites. For details, see Note S1.

Upon calibrating the MAPS model in the same way as was proposed in the original study, we noticed a strong bias toward transversion variants, for which MAPS underestimated the real number of singletons (Figure S2). As shown in Figure 1A, this resulted in missense-level predictions of negative selection for some synonymous transversion variants. In order to reduce the bias of the model, we modified the mutability values of all three classes of synonymous variants (transversions, CpGs, and non-CpG transitions) using a square-root transformation and re-fitted the model (Figure S3). We refer to this model as TRAPS. Our model does not suffer from the mutability bias observed in MAPS (Figure 1B) but shows similar evidence of deleteriousness through the enrichment of lower-frequency variants across all main functional categories of SNVs (Figure S4).

To test the performance of TRAPS, we calculated TRAPS for all synonymous variants in the ClinVar database (v. "2019-07," available as part of gnomAD v.2). Due to the limited number of synonymous variants in this database, we combined the "likely pathogenic" and "pathogenic" categories as well as the "likely benign" and "benign" categories, and we did not exclude any variants based on their review status. Figure S5 shows that according to TRAPS, pathogenic synonymous SNVs in ClinVar are on average more deleterious than synonymous VUSs (variants of uncertain significance), which are, in turn, more deleterious than benign synonymous SNVs, as expected. The difference in TRAPS scores was calculated using benign synonymous SNVs as a reference.

We also calculated TRAPS for variants in the 30% most constrained genes. To that end, we used a metric of intolerance called LOEUF, which is based on the deficit of pLoF variants in a gene.[27]

Disentangling the biological processes driving purifying selection on synonymous variants

To study the relative importance of different biological processes in driving negative selection on synonymous variants, we tested a number of potential predictors using the least absolute shrinkage and selection operator (LASSO) procedure to find variables explaining most of the variance in TRAPS. The initial list of variables included the induced change in tAI and CSC scores, to evaluate the contribution of the possibly disrupted translational efficiency and mRNA stability; the total number of codons encoding the affected amino acid, to test the hypothesis that selection on optimality-reducing variants may be not as strong in amino acids encoded by multiple codons; variant's mutability, to assess the performance of the mutability correction procedure; and change in GC, as we hypothesized that due to the high variability in GC content across genes, an imbalance in G and C bases that some synonymous variants create may contribute to higher levels of selection.

Conservation analysis

GERP (genomic evolutionary rate profiling) is a metric used for conservation analysis that can also be used as a measure of gene intolerance to SNVs.[32] In a nutshell, GERP compares evolutionary rates between positions in a reference genome to other species. It quantifies so-called "substitution deficits," that is, those substitutions that would have occurred in a DNA element if that element were neutral but did not occur because the element was under functional constraint. In other words, GERP identifies constrained loci in multiple sequence alignments by comparing the level of substitution observed to that expected if there was no functional constraint. Positive GERP scores represent highly conserved positions, while negative scores represent highly variable positions.

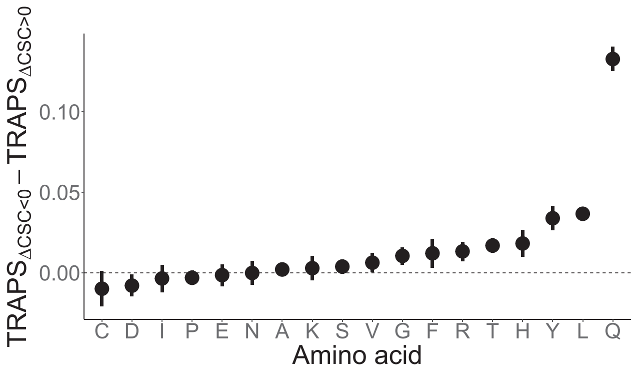

Figure 2. The intensity of the negative selection acting on optimality-reducing and optimality-increasing synonymous variants
Each dot represents the difference between TRAPS scores of optimality-reducing ($\Delta CSC < 0$) and optimality-increasing ($\Delta CSC > 0$) variants in the corresponding amino acid. Error bars are 95% binomial confidence intervals. Confidence intervals for some groups of variants are small and, therefore, not visible.

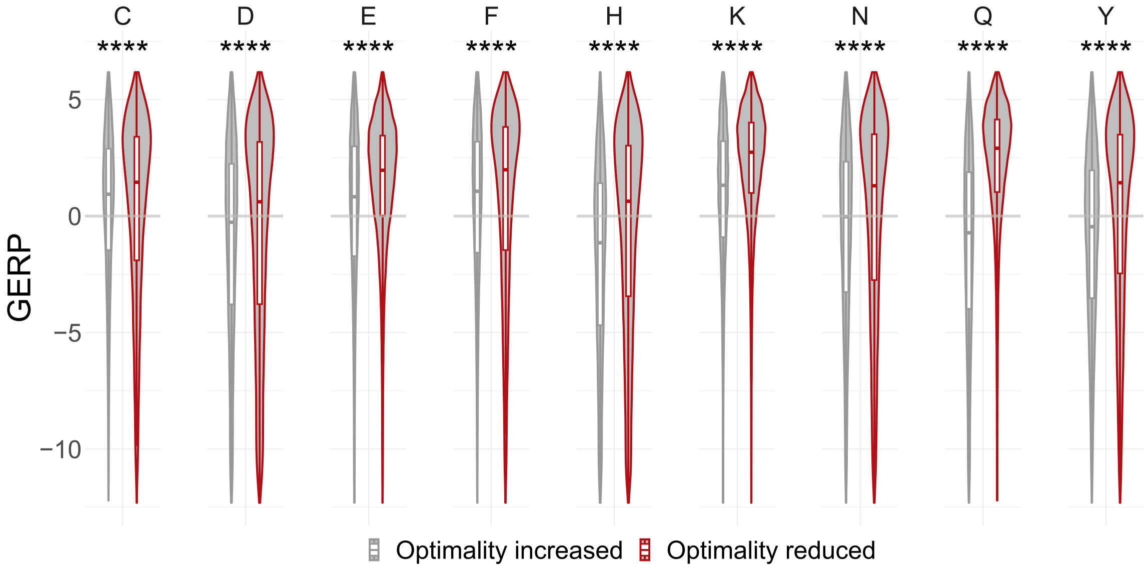

Figure 3. Distributions of GERP scores for optimality-reducing and optimality-increasing synonymous variants in two-codon amino acids
Higher positive scores indicate stronger constraint in the corresponding loci. Asterisks indicate Bonferroni-adjusted p values lower than 0.0001 (one-sided Mann-Whitney-Wilcoxon test).

A particular example of GERP scores is GERP++ scores, which can be used as estimates of evolutionary constraint for each genomic position across the mammalian lineage. We used GERP++ scores to study the relationship between the effects of synonymous SNVs on codon optimality and conservation.

Splicing

To test whether synonymous SNVs with high potential for disrupting splicing are subject to stronger negative selection, we used precomputed delta scores from SpliceAI for synonymous variants outside canonical splice sites.[33] We also investigated the synonymous variants within and outside of splicing hotspot exons, that is, exons with high susceptibility to having their splicing levels affected by internal SNVs.[34]

Variant effect predictors

CADD (combined annotation-dependent depletion) is an effect prediction tool based on a logistic regression model that scores the deleteriousness of insertions or deletions (indels) and all types of SNVs in the human genome by integrating into one metric both conservation-based information and different functional annotations.[35]

In contrast, synVep presents an alternative approach to the estimation of pathogenicity of variants, as it does not explicitly use conservation.[36] Being designed specifically for synonymous variants, synVep is a gradient-boosting model trained on existing data from gnomAD, as well as on variants that are possible but not yet observed and variants incompatible with life and, therefore, “unobservable.”

To study the concordance between TRAPS and these pathogenicity predictors, we calculated TRAPS scores for each quartile of CADD and synVep distributions.

Results

Optimality-reducing synonymous variants are subject to stronger negative selection

Using our TRAPS model, we discovered that CSC-defined optimality-reducing synonymous variants tend to be subject to stronger negative selection than optimality-increasing ones (Figure 2). We also found that CSC is negatively correlated with the minimum free energy (MFE) metric reported by the ViennaRNA software package,[37] a set of standalone programs and libraries used for prediction and analysis of RNA secondary structures, as shown in Figure S6.

In order to confirm the selective pressure against optimality-reducing variants using a metric orthogonal to MAPS, we analyzed the distributions of GERP scores for loci corresponding to those variants. To strengthen the dichotomy, we focused our analysis on two-codon amino acids. In line with our findings regarding TRAPS values, we found that optimality-reducing synonymous variants correspond to loci with high rates of rejected single-nucleotide substitutions as shown in Figure 3 and Table S1. This indicates that variants reducing optimality tend to occur at those loci that are evolutionary constrained against any genetic variation. For two-codon amino acids, this implies a particularly strong preference for one codon (more optimal) but not the other (less optimal).

In both analyses, glutamine demonstrated the highest divergence in its two groups of variants. Furthermore, we found that, given the extreme codon usage bias in glutamine (Figure 4A), its optimality-increasing variants are oversaturated in gnomAD (Figure 4B). For all other two-codon amino acids, all of which have a rather modest codon usage bias, optimality-reducing variants tend to exhaust the genome-wide pool of optimal codons quicker than their optimality-increasing counterparts exhaust the pool of nonoptimal codons. In contrast, glutamine’s optimality-reducing variants are depleted compared to the expected level.

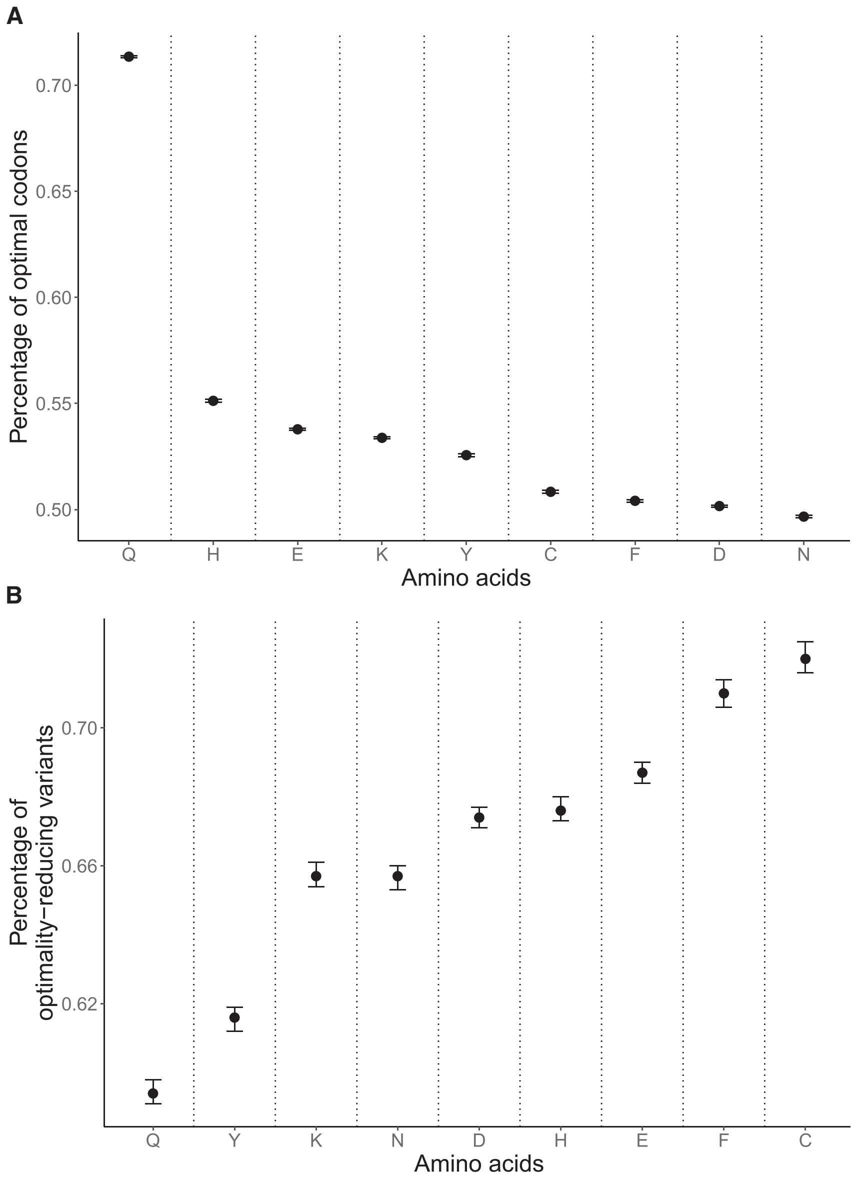

Figure 4. Relationship between codon usage and percentage of optimality-reducing variants by amino acid
(A) Average codon usage bias in the human genome calculated using codon usage statistics from the CoCoPUTs database. Error bars are 95% binomial confidence intervals (Wilson score method).
(B) Percentage of optimality-reducing variants in gnomAD for all two-codon amino acids. Error bars are 95% binomial confidence intervals (Wilson score method).

Moreover, TRAPS scores calculated for variants in constrained genes were elevated compared to TRAPS scores in all genes, and this increase was positive across all amino acids regardless of the direction of optimality change (Figure S7). This indicates that, as expected, synonymous variants are affected by the background selection that operates in highly constrained genes. It also shows that these genes are less tolerant to synonymous variants since they could alter mRNA stability and therefore impact gene dosage.

tRNA availability and mRNA stability drive the intensity of purifying selection on synonymous variants

By using a LASSO procedure, we were able to find that some of the key predictors of the purifying selection captured by TRAPS include the gain in tRNA availability (tAI) and mRNA stability (CSC), as well as the resulting change in GC content (Figure 5). These three variables helped explain a total of 21% variance in TRAPS scores (see Note S2 and Table S2). Besides, we observed a strong positive correlation between changes in CSC and GC content (Figure S8), which supports the hypothesis that mRNA stability is largely driven by stacks of G and C nucleotides, which provide more strength to the structure than any other consecutive base pairs.[38,39] Apart from tAI, CSC, and GC content, we also included the total number of codons (codon degeneracy) and averaged mutability as input variables for LASSO, both of which were subsequently removed, indicating the robustness of the mutability correction procedure.

TRAPS scores for synonymous variants are correlated with CADD and synVep predictions

Upon the inspection of the distribution of TRAPS scores for CADD and synVep quartiles, we found that TRAPS is mostly well correlated with both CADD and synVep, as can be seen in Figure 6. Although the first two quartiles of the synVep distribution are an exception to this trend, we speculate that this might be due to the fact that synVep's least-deleterious variants do not possess a strong enough signal that could be captured by TRAPS.

Differences in selection against splice-altering synonymous variants

We found that some classes of synonymous variants with high potential for disrupting splicing are subject to stronger negative selection than others. Specifically, in our dataset, variants outside splice sites with SpliceAI scores at or above 0.5 predicted to result in a splice-site loss exhibited much higher TRAPS scores than splice-site-gain variants, with the values for losses being close to pLoF levels (Figures 7 and S4). Importantly, the loci affected

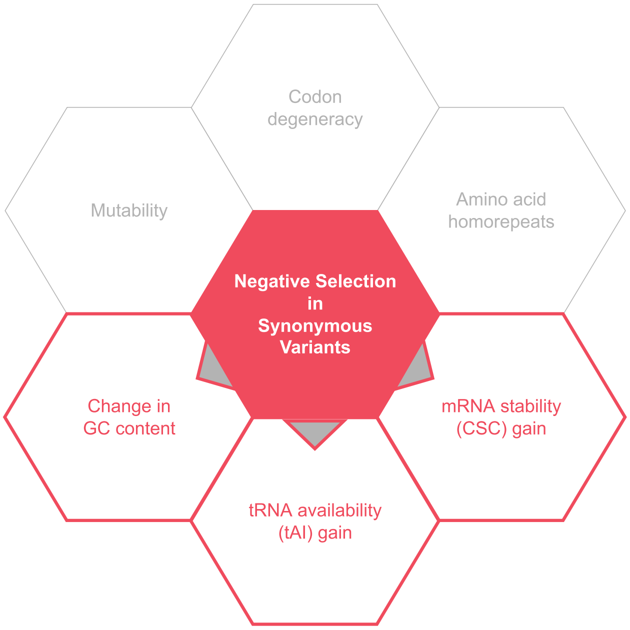

Figure 5. Results of the LASSO procedure showing some of the predictors of the negative selection captured by TRAPS in synonymous variants
Those variables that were tested but not included in the final model are dimmed.

by those high-scoring synonymous splice-site losses were shifted away from the exon boundary, as shown in Figure S9. This not only confirms that our filtering procedure effectively removed variants in canonical splice sites but also suggests that the SpliceAI-predicted effect in the remaining variants is largely due to their exonic potential to disrupt splicing. Incidentally, we observed no statistically significant difference between optimality-reducing and optimality-increasing synonymous variants in and outside of splicing hotspot exons (Figure S10).

Discussion

In this work, we attempted to examine the extent of the purifying selection acting on synonymous SNVs and disentangle the relative contribution of RNA stability and tRNA availability in this process. Based on our TRAPS model, negative selection on synonymous SNVs is dependent not only on the identity of the affected amino acid but also on a number of locally induced changes to the properties of the coding sequence. Our results agree with the previously reported evidence of selection on synonymous codons observed in some of the most state-of-the-art population genomics databases like the UK Biobank[22,23] and go deeper into demonstrating what shapes this selection.

The negative-selection approach that was used in this work is different from the popular machine learning methods that are being increasingly used in numerous variant pathogenicity predictors, some of which are designed specifically for the analysis of synonymous variants (SPI,[40] usDSM,[41] SiLVA[42]). With the exception of the above-mentioned tool synVep, the vast majority of currently available pathogenicity predictors tend to employ methods like random forest (usDSM, SiLVA) and gradient boosting (SPI) to train a model on a set of features using datasets with variants pre-labeled according to their supposed deleteriousness. This results in highly convoluted prediction models, especially in ensemble-based methods,[43] at the core of which is the notorious problem of overreliance on training data. The latter is especially true of synonymous variants, as it is almost impossible to know the individual pathogenicity of such variants from experimental analyses. In contrast, here we started with the population-level selection forces as a proxy for pathogenicity in the aim to unfold it through a set of potential predictors using the LASSO framework.

Compared with the analysis of selection on genetic variants belonging to different types of variation (e.g., all pLoF versus all missense), for which MAPS is an appropriate choice, we found that MAPS may be unsuitable for studying selection patterns within individual classes of SNVs. Due to the comparatively small size of most of our subsets of synonymous variants, we observed that the estimated degree of selection based on MAPS values in those subsets was highly sensitive to the model fit. Therefore, we modified the calibration method used in MAPS to create TRAPS, a model that provides more reliable estimates of negative selection in smaller sets of variants.

Consistent with the literature,[21] we found that CSC-defined optimality-reducing synonymous variants are subject to stronger negative selection than their optimality-increasing counterparts. As expected, the TRAPS scores of optimality-reducing synonymous variants were higher than those of optimality-increasing variants (see Figure S11), though they were still lower than previously reported scores for missense and pLoF variants.[27] Also, we found that the average negative-selection scores for missense, pLoF, and other classes of variants, with the exception of synonymous variants, which are used for calibration and therefore are always at zero, are all slightly lower in TRAPS (see Figure S4), which is not surprising given that the mutability bias in MAPS makes it overestimate deleteriousness.

We argue that CSC is conceptually the opposite of the MFE metric from the ViennaRNA software package,[37] as the "stability" of mRNA transcripts in the definition of CSC is directly related to the amount of free (destabilizing) energy that these transcripts have. According to the theory behind MFE, transcripts exhibiting significant amounts of free energy tend to be weaker and have shorter lifespans, thus producing less protein; however, those exhibiting little free energy, too, produce less protein, as their increased stability may hinder translation.[44]

Consistent with the observations made in yeast,[20] we also found a degree of similarity between human CSC

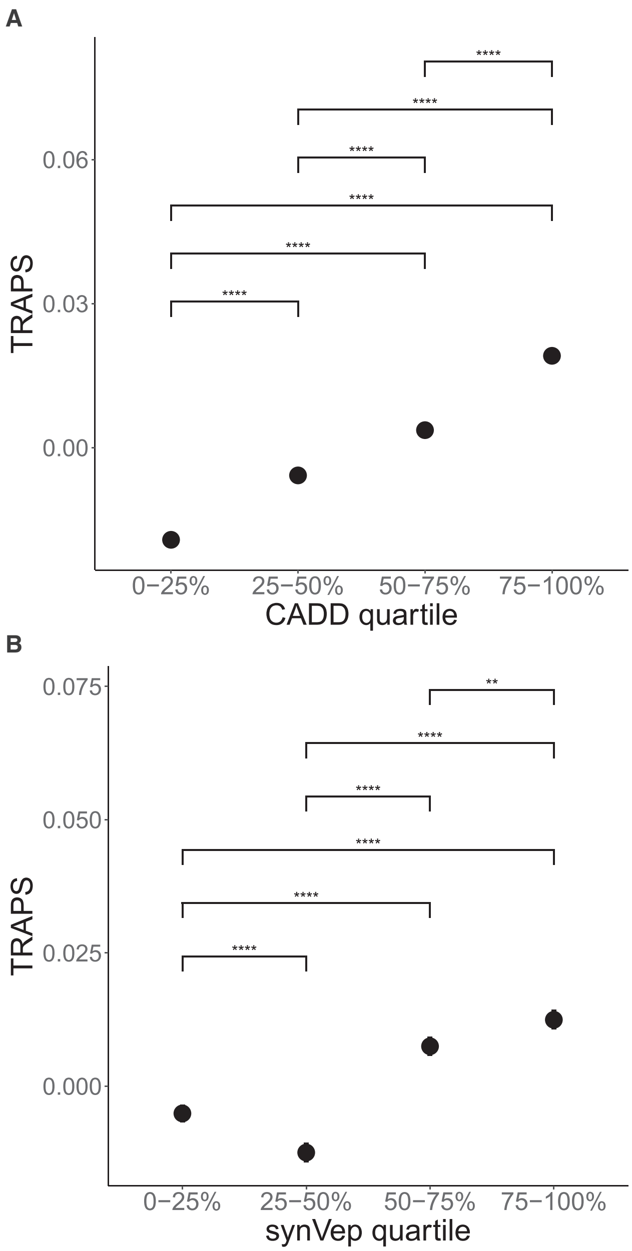

Figure 6. Correlation between TRAPS and pathogenicity predictors CADD and synVep
In both TRAPS-CADD (A) and TRAPS-synVep (B) correlation plots, unannotated synonymous variants are not included. Error bars are 95% binomial confidence intervals. Bonferroni-adjusted p values are shown for each comparison (Welch modified two-sample t test): *$p < 0.05$, **$p < 0.01$, ***$p < 0.001$, ****$p < 0.0001$, and ns, not significant.

and tAI metrics. Notably, in the context of two-codon amino acids, CSC and the tAI always agree. That is, in histidine, lysine, and other two-codon amino acids, the optimal codon is optimal according to both the tAI and CSC. In amino acids encoded by more than two codons, however, there is no such consistency. Nevertheless, we would like to note that the tAI is not a perfect proxy for tRNA abundance, as it does not account for possible tRNA collisions, for example. Furthermore, the tRNA copy number that is used for the calculation of the tAI is not always correlated with tRNA levels in higher eukaryotes.[45] More important still, tRNA expression is tissue specific.

We hypothesize that the increase in TRAPS scores that we observe for synonymous variants in constrained genes can be attributed to the joint action of background selection and the increased negative selection operating specifically on these variants. Regardless of the origins of this selection, the fact that TRAPS scores are elevated in these genes indicates that the metric captures the right signal.

One of the key findings in our work is that concerning the amino acid glutamine. Although we found that glutamine was consistently an outlier in our results, we did not identify what factors could be attributed to this observation. It has been shown that a number of human neurodegenerative disorders are caused by the abnormal expansion of the “CAG” codon in poly-glutamine homorepeats.[46] In light of the recently proposed theory of possible counterselection against the “CAG” codon in homorepeats beyond the optimal length,[47] we calculated the averaged TRAPS scores for synonymous variants in short and long stretches of identical amino acid residues. However, the obtained results did not help us to further improve our predictor model nor explain the trends that we noticed in glutamine.

Although we observed high divergence in the TRAPS and GERP scores of glutamine’s optimality-reducing and optimality-increasing variants, the absolute yield in optimality for its “CAG”/“CAA” codon transitions is rather moderate compared to, for example, cysteine (“TGT”/“TGC”), as can be seen in Figure S12. Interestingly, in a recent study on the pathogenicity of synonymous variants, where the single-nucleotide context level was analyzed, the “G>A” context was consistently not following the trends observed in other, similar contexts.[40] Given that glutamine’s “CAG”-to-“CAA” codon transitions fall into this exact group, this further strengthens our hypothesis that SNVs in glutamine might present a special case in the context of synonymous variants, and further research on this is required.

Finally, we found that exonic, synonymous SNVs outside splice sites that are predicted to disrupt splicing (based on SpliceAI delta scores) undergo strong negative selection. Interestingly, we also found that variants predicted to cause donor and acceptor splice-site losses show higher TRAPS scores than splice-site gains. Although this indicates that splice-site losses could undergo stronger negative selection, it might also highlight that these variants are easier to predict. This agrees with the observation that the validation rate is higher for donor/acceptor loss variants than for donor/acceptor gain variants.[33] We suggest that giving more “weight” to high-scoring splice-site losses could help achieve higher accuracy of variant pathogenicity predictors. Overall, we hope that our findings with respect to the effects of synonymous variants on splicing

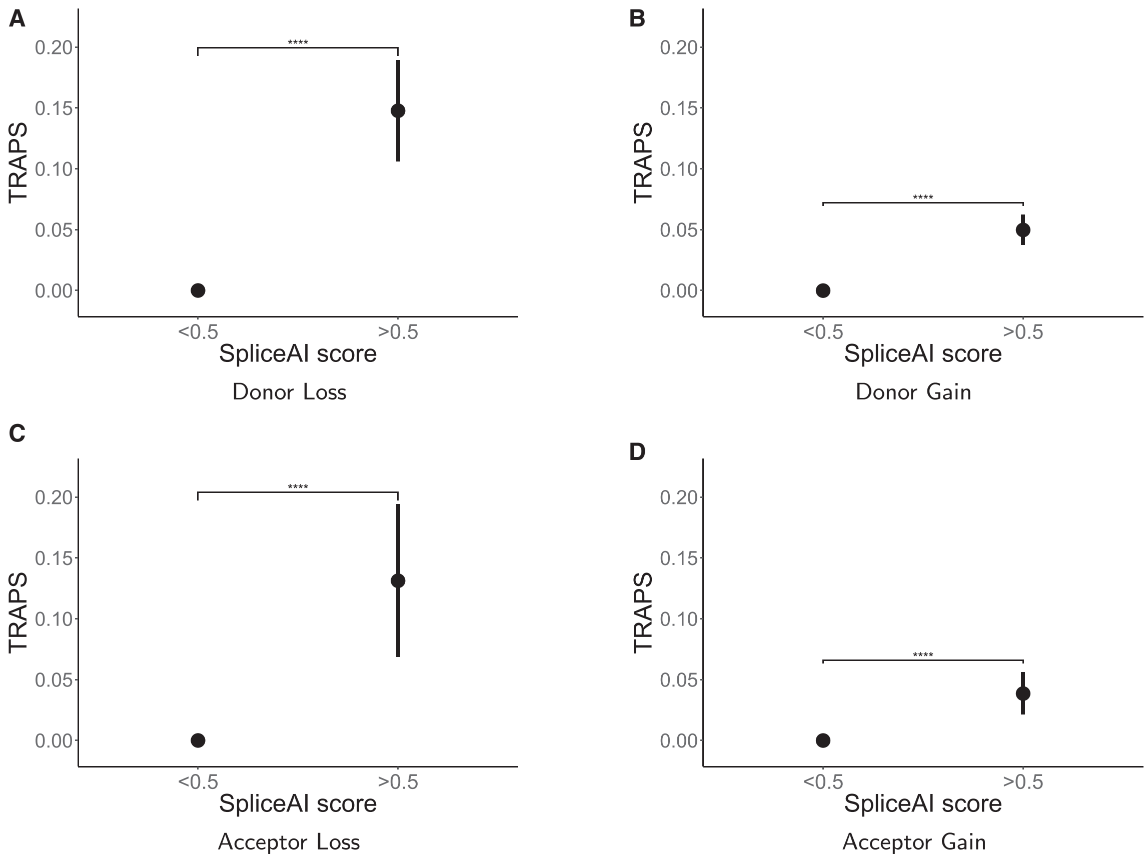

Figure 7. TRAPS scores for synonymous variants by predicted effect on splicing
Variants with low (SpliceAI delta score lower than 0.5) and high (SpliceAI delta score at or above 0.5) potential for disrupting splicing are compared for "donor loss" (A), "donor gain" (B), "acceptor loss" (C), and "acceptor gain" (D) prediction classes. Unannotated synonymous variants are not included. Error bars are 95% binomial confidence intervals. Confidence intervals for variants with SpliceAI score below 0.5 are small and, therefore, not visible. A p value is shown for each group (Welch modified two-sample t test): *p $<$ 0.05, **p $<$ 0.01, ***p $<$ 0.001, ****p $<$ 0.0001, and ns, not significant.

will help improve the performance of some of the above-mentioned predictor tools.

Conclusion

We believe that the results of this work will help to further elucidate the role of synonymous SNVs in health and disease and, in particular, will aid in variant prioritization for finding the true causes of genetic disorders. We hope that some of the aspects of synonymous SNVs that we attempted to cover in this study in the context of negative selection will guide the developers of pathogenicity predictors toward even more advanced algorithms.

Data and code availability

The code generated during this study is available at https://github.com/VCCRI/sSNVs.

Supplemental information

Supplemental information can be found online at https://doi.org/10.1016/j.xhgg.2024.100262.

Acknowledgments

This work was supported by the NSW state government (E.G.), the NSW Health Early-Mid Career Fellowship (E.G.), and the National Health and Medical Research Council (2018360) (E.G.).

Declaration of interests

The authors declare no competing interests.

Received: August 21, 2023
Accepted: January 1, 2024

Web resources

gnomAD, https://gnomad.broadinstitute.org.

References

1. Comeron, J.M. (2004). Selective and mutational patterns associated with gene expression in humans: Influences on synonymous composition and intron presence. Genetics *167*, 1293–1304.

2. Lavner, Y., and Kotlar, D. (2005). Codon bias as a factor in regulating expression via translation rate in the human genome. Gene *345*, 127–138.
3. Cartegni, L., Chew, S.L., and Krainer, A.R. (2002). Listening to silence and understanding nonsense: exonic mutations that affect splicing. Nat. Rev. Genet. *3*, 285–298.
4. Pagani, F., Raponi, M., and Baralle, F.E. (2005). Synonymous mutations in CFTR exon 12 affect splicing and are not neutral in evolution. Proc. Natl. Acad. Sci. USA *102*, 6368–6372.
5. Parmley, J.L., Chamary, J.V., and Hurst, L.D. (2006). Evidence for purifying selection against synonymous mutations in mammalian exonic splicing enhancers. Mol. Biol. Evol. *23*, 301–309.
6. Savisaar, R., and Hurst, L.D. (2018). Exonic splice regulation imposes strong selection at synonymous sites. Genome Res. *28*, 1442–1454.
7. Brest, P., Lapaquette, P., Souidi, M., Lebrigand, K., Cesaro, A., Vouret-Craviari, V., Mari, B., Barbry, P., Mosnier, J.F., Hébuterne, X., et al. (2011). A synonymous variant in IRGM alters a binding site for miR-196 and causes deregulation of IRGM-dependent xenophagy in Crohn's disease. Nat. Genet. *43*, 242–245.
8. Bartoszewski, R.A., Jablonsky, M., Bartoszewska, S., Stevenson, L., Dai, Q., Kappes, J., Collawn, J.F., and Bebok, Z. (2010). A Synonymous Single Nucleotide Polymorphism in ΔF508 CFTR Alters the Secondary Structure of the mRNA and the Expression of the Mutant Protein. J. Biol. Chem. *285*, 28741–28748.
9. Duan, J., Wainwright, M.S., Comeron, J.M., Saitou, N., Sanders, A.R., Gelernter, J., and Gejman, P.V. (2003). Synonymous mutations in the human dopamine receptor D2 (DRD2) affect mRNA stability and synthesis of the receptor. Hum. Mol. Genet. *12*, 205–216.
10. Buhr, F., Jha, S., Thommen, M., Mittelstaet, J., Kutz, F., Schwalbe, H., Rodnina, M.V., and Komar, A.A. (2016). Synonymous codons direct cotranslational folding toward different protein conformations. Mol. Cell *61*, 341–351.
11. Karakostis, K., Vadivel Gnanasundram, S., López, I., Thermou, A., Wang, L., Nylander, K., Olivares-Illana, V., and Fåhraeus, R. (2019). A single synonymous mutation determines the phosphorylation and stability of the nascent protein. J. Mol. Cell Biol. *11*, 187–199.
12. Kudla, G., Murray, A.W., Tollervey, D., and Plotkin, J.B. (2009). Coding-Sequence Determinants of Gene Expression in Escherichia coli. Science *324*, 255–258.
13. Yu, C.-H., Dang, Y., Zhou, Z., Wu, C., Zhao, F., Sachs, M.S., and Liu, Y. (2015). Codon usage influences the local rate of translation elongation to regulate co-translational protein folding. Mol. Cell *59*, 744–754.
14. Jiang, Y., Neti, S.S., Sitarik, I., Pradhan, P., To, P., Xia, Y., Fried, S.D., Booker, S.J., and O'Brien, E.P. (2023). How synonymous mutations alter enzyme structure and function over long timescales. Nat. Chem. *15*, 308–318.
15. Rosenberg, A.A., Marx, A., and Bronstein, A.M. (2022). Codon-specific Ramachandran plots show amino acid backbone conformation depends on identity of the translated codon. Nat. Commun. *13*, 2815.
16. Sharp, P.M., and Li, W.-H. (1987). The codon adaptation index-a measure of directional synonymous codon usage bias, and its potential applications. Nucleic Acids Res. *15*, 1281–1295.
17. Sauna, Z.E., and Kimchi-sarfaty, C. (2011). Understanding the contribution of synonymous mutations to human disease. Nat. Rev. Genet. *12*, 683–691.
18. dos Reis, M., Savva, R., and Wernisch, L. (2004). Solving the riddle of codon usage preferences: A test for translational selection. Nucleic Acids Res. *32*, 5036–5044.
19. Tuller, T., Carmi, A., Vestsigian, K., Navon, S., Dorfan, Y., Zaborske, J., Pan, T., Dahan, O., Furman, I., and Pilpel, Y. (2010). An evolutionarily conserved mechanism for controlling the efficiency of protein translation. Cell *141*, 344–354.
20. Presnyak, V., Alhusaini, N., Chen, Y.-H., Martin, S., Morris, N., Kline, N., Olson, S., Weinberg, D., Baker, K.E., Graveley, B.R., and Coller, J. (2015). Codon optimality is a major determinant of mRNA stability. Cell *160*, 1111–1124.
21. Dhindsa, R.S., Copeland, B.R., Mustoe, A.M., and Goldstein, D.B. (2020). Natural selection shapes codon usage in the human genome. Am. J. Hum. Genet. *107*, 83–95.
22. Dhindsa, R.S., Wang, Q., Vitsios, D., Burren, O.S., Hu, F., DiCarlo, J.E., Kruglyak, L., MacArthur, D.G., Hurles, M.E., and Petrovski, S. (2022). A minimal role for synonymous variation in human disease. Am. J. Hum. Genet. *109*, 2105–2109.
23. Halldorsson, B.V., Eggertsson, H.P., Moore, K.H.S., Hauswedell, H., Eiriksson, O., Ulfarsson, M.O., Palsson, G., Hardarson, M.T., Oddsson, A., Jensson, B.O., et al. (2022). The sequences of 150,119 genomes in the UK Biobank. Nature *607*, 732–740.
24. Wu, Q., Medina, S.G., Kushawah, G., DeVore, M.L., Castellano, L.A., Hand, J.M., Wright, M., and Bazzini, A.A. (2019). Translation affects mRNA stability in a codon-dependent manner in human cells. Elife *8*, e45396.
25. Drummond, D.A., and Wilke, C.O. (2008). Mistranslation-induced protein misfolding as a dominant constraint on coding-sequence evolution. Cell *134*, 341–352.
26. Alexaki, A., Kames, J., Holcomb, D.D., Athey, J., Santana-Quintero, L.V., Lam, P.V.N., Hamasaki-Katagiri, N., Osipova, E., Simonyan, V., Bar, H., et al. (2019). Codon and codon-pair usage tables (CoCoPUTs): facilitating genetic variation analyses and recombinant gene design. J. Mol. Biol. *431*, 2434–2441.
27. Karczewski, K.J., Francioli, L.C., Tiao, G., Cummings, B.B., Alföldi, J., Wang, Q., Collins, R.L., Laricchia, K.M., Ganna, A., Birnbaum, D.P., et al. (2020). The mutational constraint spectrum quantified from variation in 141,456 humans. Nature *581*, 434–443.
28. Lek, M., Karczewski, K.J., Minikel, E.V., Samocha, K.E., Banks, E., Fennell, T., O'Donnell-Luria, A.H., Ware, J.S., Hill, A.J., Cummings, B.B., et al. (2016). Analysis of protein-coding genetic variation in 60,706 humans. Nature *536*, 285–291.
29. Samocha, K.E., Robinson, E.B., Sanders, S.J., Stevens, C., Sabo, A., McGrath, L.M., Kosmicki, J.A., Rehnström, K., Mallick, S., Kirby, A., et al. (2014). A framework for the interpretation of de novo mutation in human disease. Nat. Genet. *46*, 944–950.
30. Karczewski, K.J., and Martin, A.R. (2020). Analytic and translational genetics. Annu. Rev. Biomed. Data Sci. *3*, 217–241.
31. McLaren, W., Gil, L., Hunt, S.E., Riat, H.S., Ritchie, G.R.S., Thormann, A., Flicek, P., and Cunningham, F. (2016). The Ensembl Variant Effect Predictor. Genome Biol. *17*, 122.
32. Davydov, E.V., Goode, D.L., Sirota, M., Cooper, G.M., Sidow, A., and Batzoglou, S. (2010). Identifying a high fraction of the human genome to be under selective constraint using GERP++. PLoS Comput. Biol. *6*, 1–13.
33. Jaganathan, K., Kyriazopoulou Panagiotopoulou, S., McRae, J.F., Darbandi, S.F., Knowles, D., Li, Y.I., Kosmicki, J.A.,

Arbelaez, J., Cui, W., Schwartz, G.B., et al. (2019). Predicting splicing from primary sequence with deep learning. Cell *176*, 535–548.e24.

34. Glidden, D.T., Buerer, J.L., Saueressig, C.F., and Fairbrother, W.G. (2021). Hotspot exons are common targets of splicing perturbations. Nat. Commun. *12*, 2756.
35. Rentzsch, P., Witten, D., Cooper, G.M., Shendure, J., and Kircher, M. (2019). CADD: predicting the deleteriousness of variants throughout the human genome. Nucleic Acids Res. *47*, D886–D894.
36. Zeng, Z., Aptekmann, A.A., and Bromberg, Y. (2021). Decoding the effects of synonymous variants. Preprint at bioRxiv.
37. Lorenz, R., Bernhart, S.H., Höner Zu Siederdissen, C., Tafer, H., Flamm, C., Stadler, P.F., and Hofacker, I.L. (2011). ViennaRNA Package 2.0. Algorithm Mol. Biol. *6*, 26.
38. Hia, F., Yang, S.F., Shichino, Y., Yoshinaga, M., Murakawa, Y., Vandenbon, A., Fukao, A., Fujiwara, T., Landthaler, M., Natsume, T., et al. (2019). Codon bias confers stability to human mRNAs. EMBO Rep. *20*, e48220.
39. Yakovchuk, P., Protozanova, E., and Frank-Kamenetskii, M.D. (2006). Base-stacking and base-pairing contributions into thermal stability of the DNA double helix. Nucleic Acids Res. *34*, 564–574.
40. Gaither, J.B.S., Lammi, G.E., Li, J.L., Gordon, D.M., Kuck, H.C., Kelly, B.J., Fitch, J.R., and White, P. (2021). Synonymous variants that disrupt messenger RNA structure are significantly constrained in the human population. GigaScience *10*, giab023.
41. Tang, X., Zhang, T., Cheng, N., Wang, H., Zheng, C.-H., Xia, J., and Zhang, T. (2021). usDSM: a novel method for deleterious synonymous mutation prediction using undersampling scheme. Briefings Bioinf. *22*, bbab247.
42. Buske, O.J., Manickaraj, A., Mital, S., Ray, P.N., and Brudno, M. (2013). Identification of deleterious synonymous variants in human genomes. Bioinformatics *29*, 1843–1850.
43. Ranganathan Ganakammal, S., and Alexov, E. (2020). An ensemble approach to predict the pathogenicity of synonymous variants. Genes *11*, 1102.
44. Plotkin, J.B., and Kudla, G. (2011). Synonymous but not the same: The causes and consequences of codon bias. Nat. Rev. Genet. *12*, 32–42.
45. Zheng, G., Qin, Y., Clark, W.C., Dai, Q., Yi, C., He, C., Lambowitz, A.M., and Pan, T. (2015). Efficient and quantitative high-throughput tRNA sequencing. Nat. Methods *12*, 835–837.
46. Fan, H.-C., Ho, L.-I., Chi, C.-S., Chen, S.-J., Peng, G.-S., Chan, T.-M., Lin, S.-Z., and Harn, H.-J. (2014). Polyglutamine (PolyQ) diseases: genetics to treatments. Cell Transplant. *23*, 441–458.
47. Mier, P., and Andrade-Navarro, M.A. (2018). Glutamine Codon Usage and polyQ Evolution in Primates Depend on the Q Stretch Length. Genome Biol. Evol. *10*, 816–825.

HGGA, Volume *5*

Supplemental information

Quantifying negative selection on synonymous variants

Mikhail Gudkov, Loïc Thibaut, and Eleni Giannoulatou

Supplemental Figures

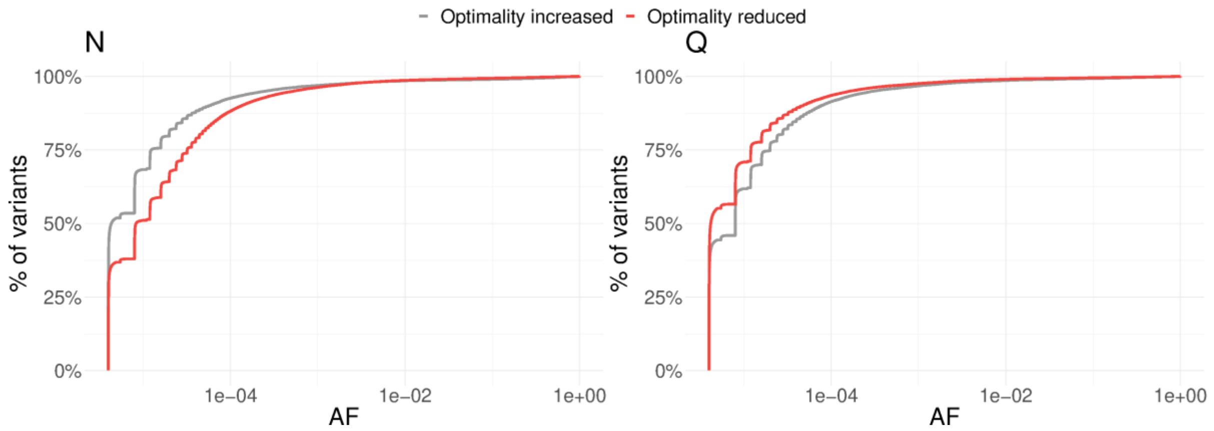

Figure S1. Example of a difference in allele frequencies of variants due to different mutability. Compared to glutamine (Q), the proportion of singletons in asparagine's (N) optimality-reducing variants (red line) is relatively low because of the contribution of the highly mutable CpGs transitions ("xxC->xxT").

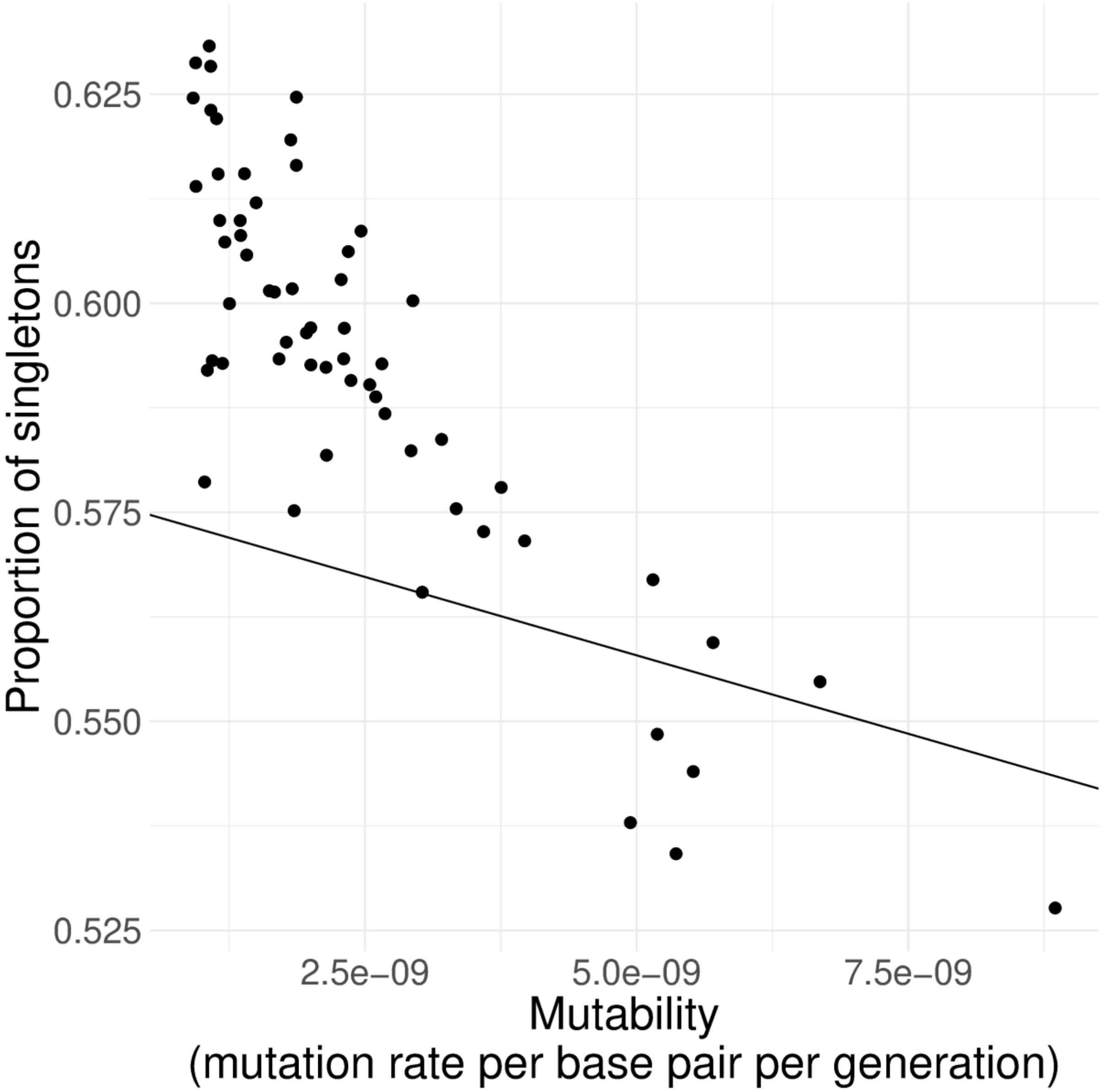

Figure S2. Bias towards synonymous transversion variants in the MAPS model. The line indicates the slope of the MAPS model calibrated on all synonymous variants (transversions, CpGs and non-CpG transitions). Each dot corresponds to a unique mutability value that a transversion variant can take.

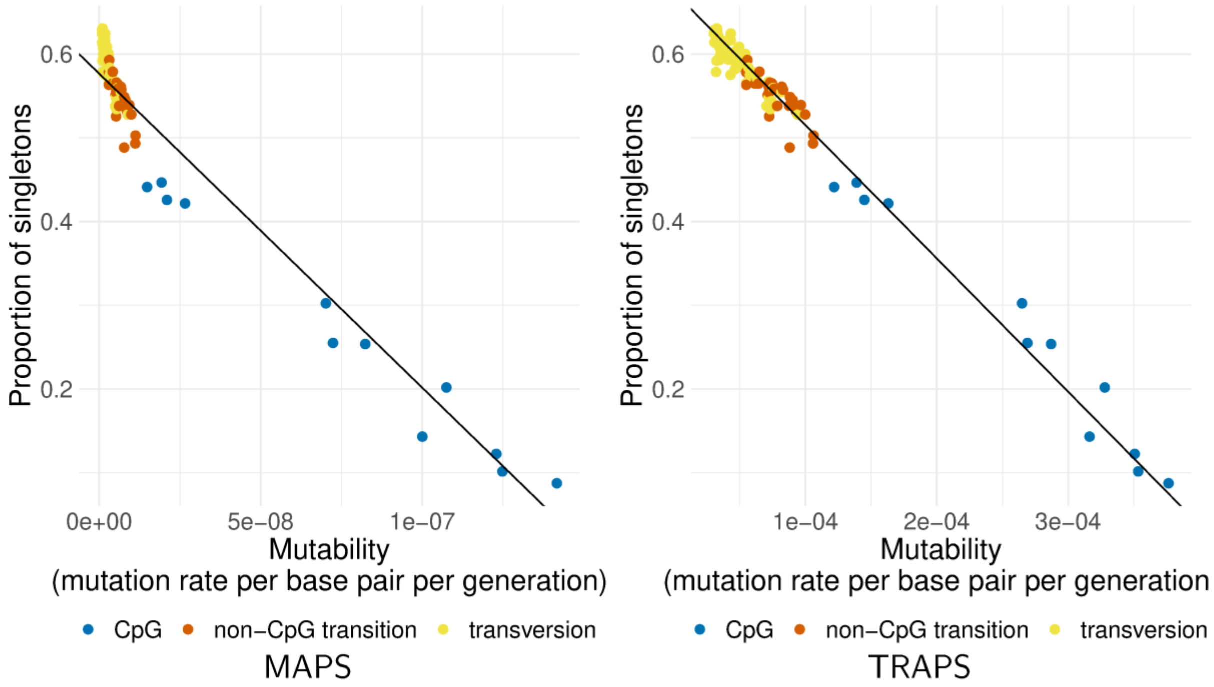

Figure S3. The effect of square-root transformation on the fit of the MAPS model of the expected number of singletons.

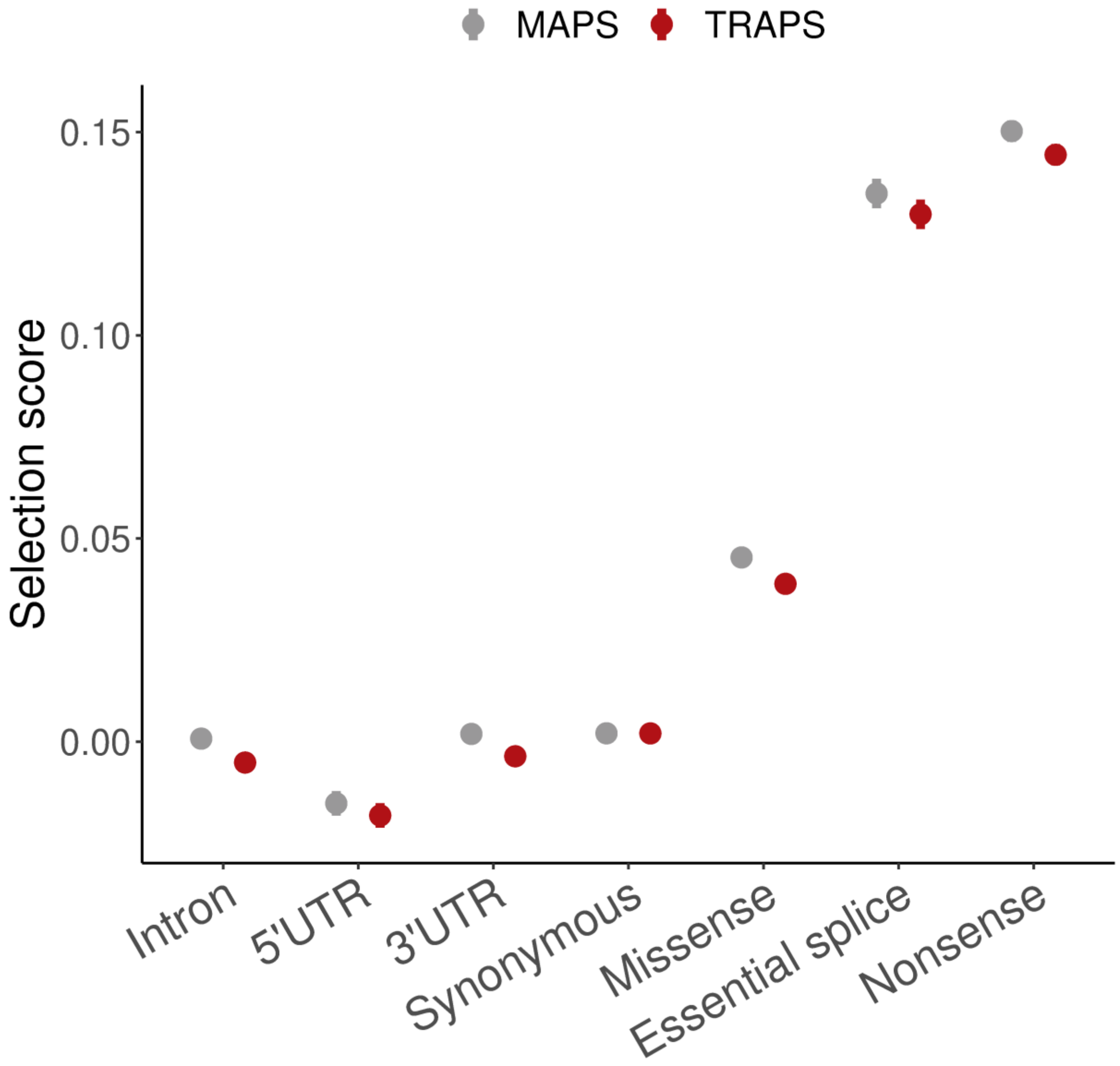

Figure S4. Estimates of negative selection in SNVs by variant class, calculated using MAPS and TRAPS. Error bars are 95% binomial confidence intervals.

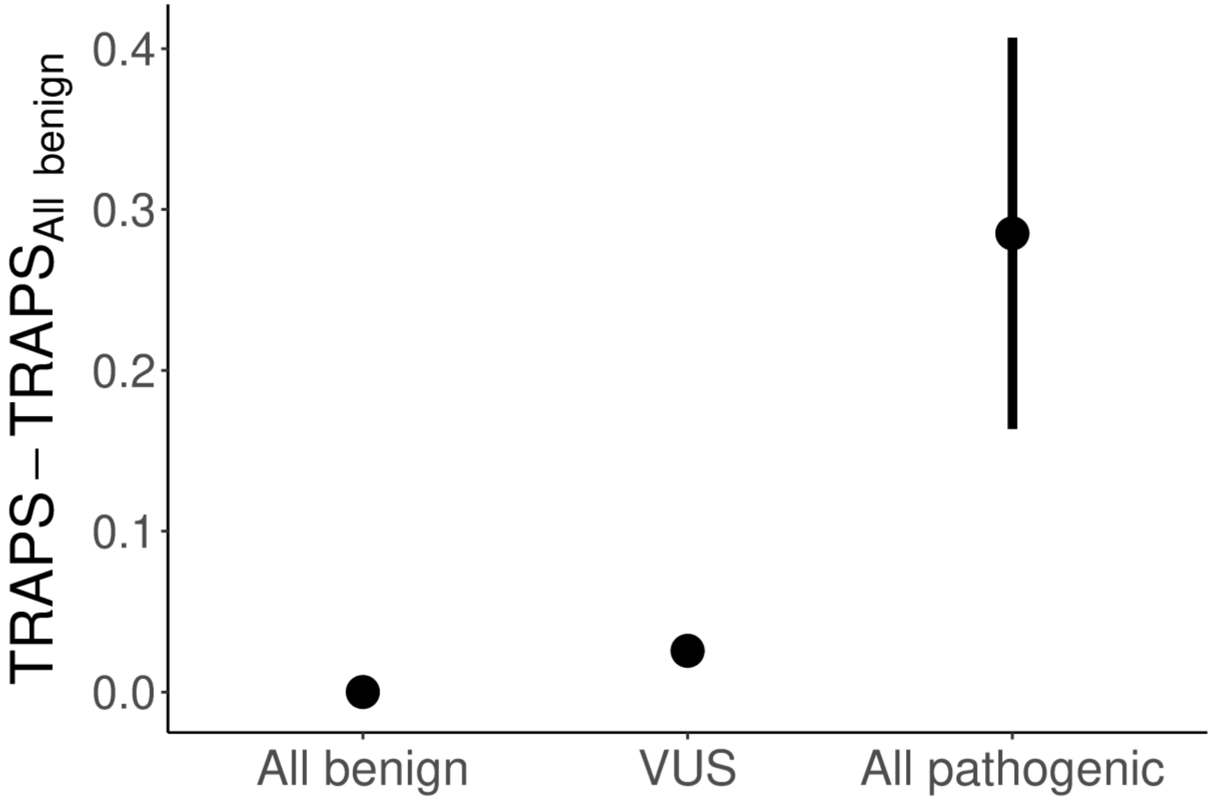

Figure S5. *Δ*TRAPS scores for synonymous variants from ClinVar (version "2019-07") by category, with "All benign" variants used as a reference. Error bars are 95% binomial confidence intervals.

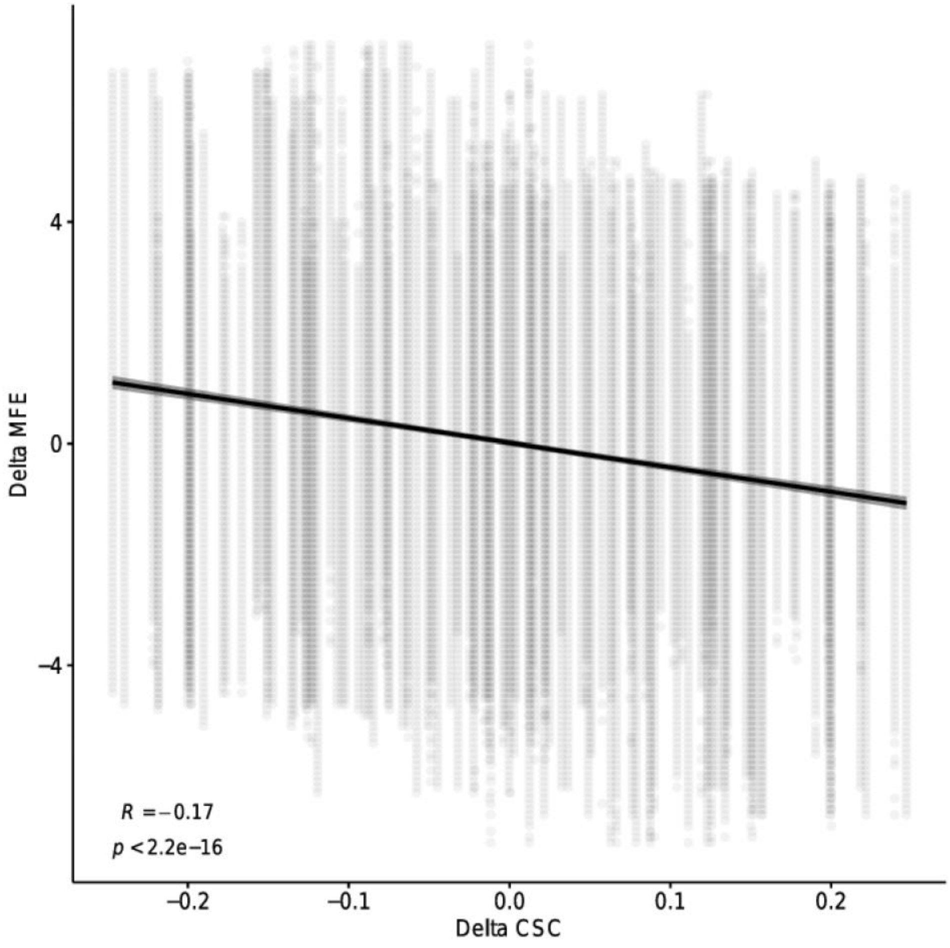

Figure S6. Correlation between ΔCSC (Codon Stability Coefficient) and ΔMFE (Minimum Free Energy), showing that positive changes in CSC (stabilising variants) tend to correspond to negative changes in Minimum Free Energy.

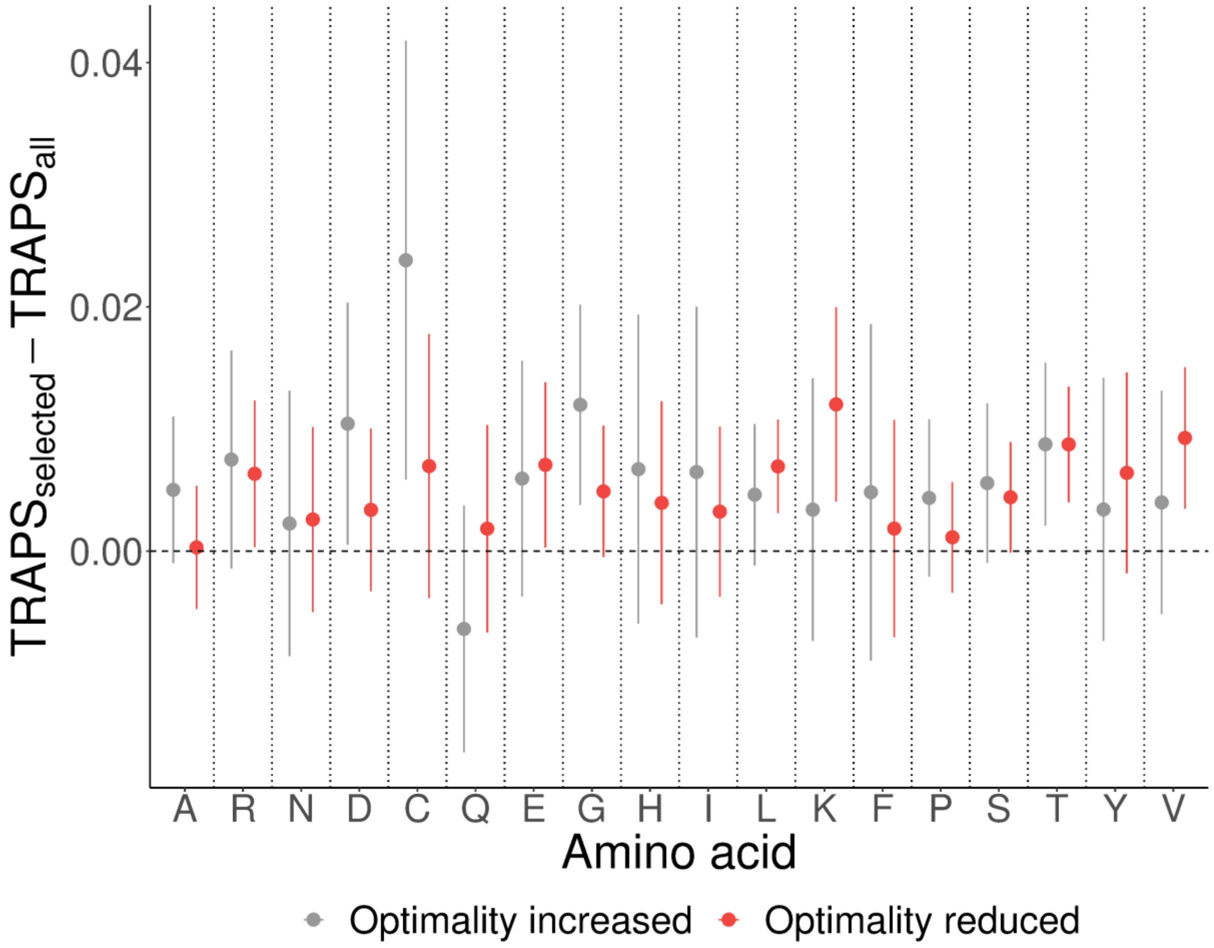

Figure S7. Difference between TRAPS scores in 30% most constrained genes (based on the LOEUF metric of intolerance) and in all genes. Error bars are 95% binomial confidence intervals.

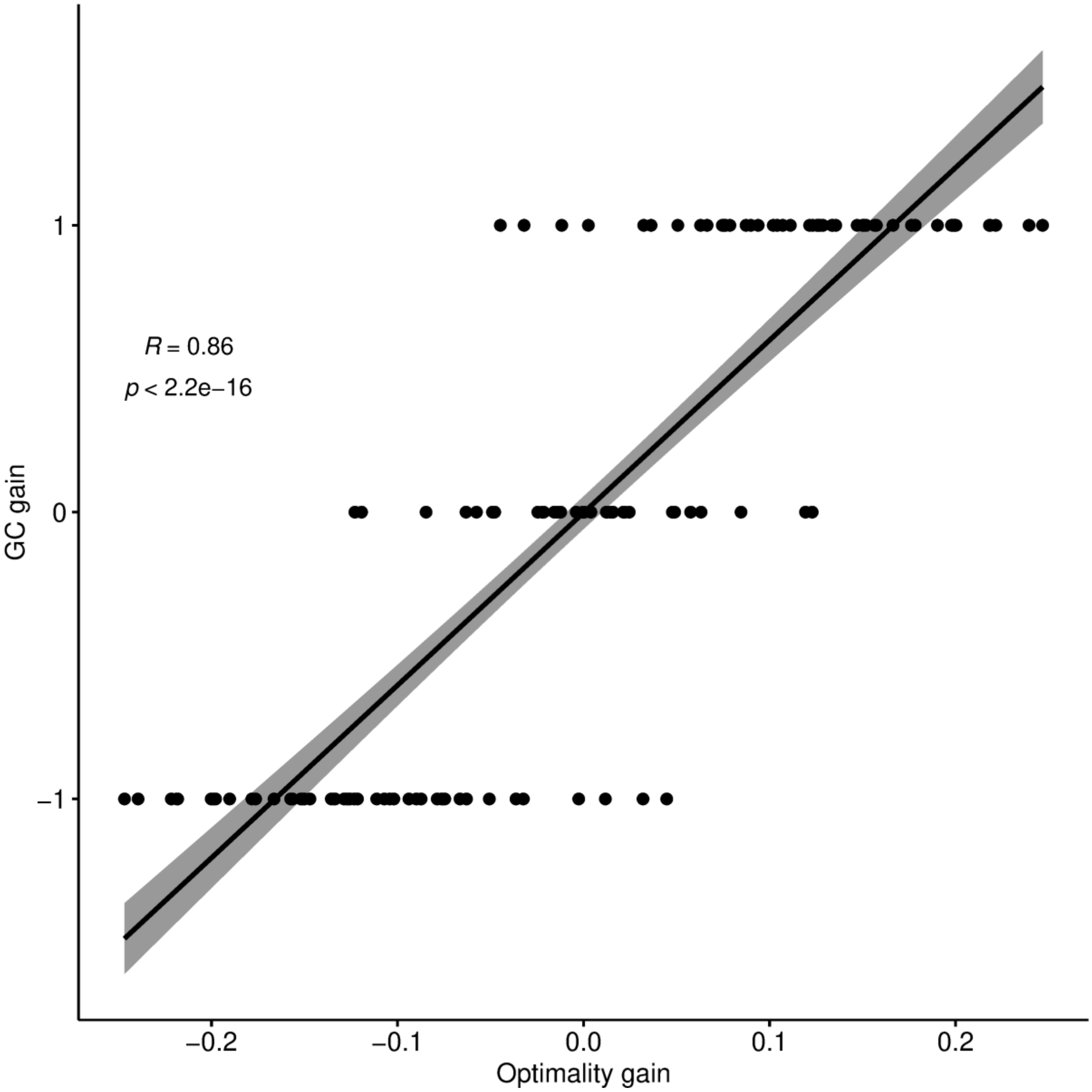

Figure S8. Correlation between optimality gain (*Δ*CSC) and change in GC content. A GC change of "-1" ("+1") indicates a G/C to A/T (A/T to G/C) substitution.

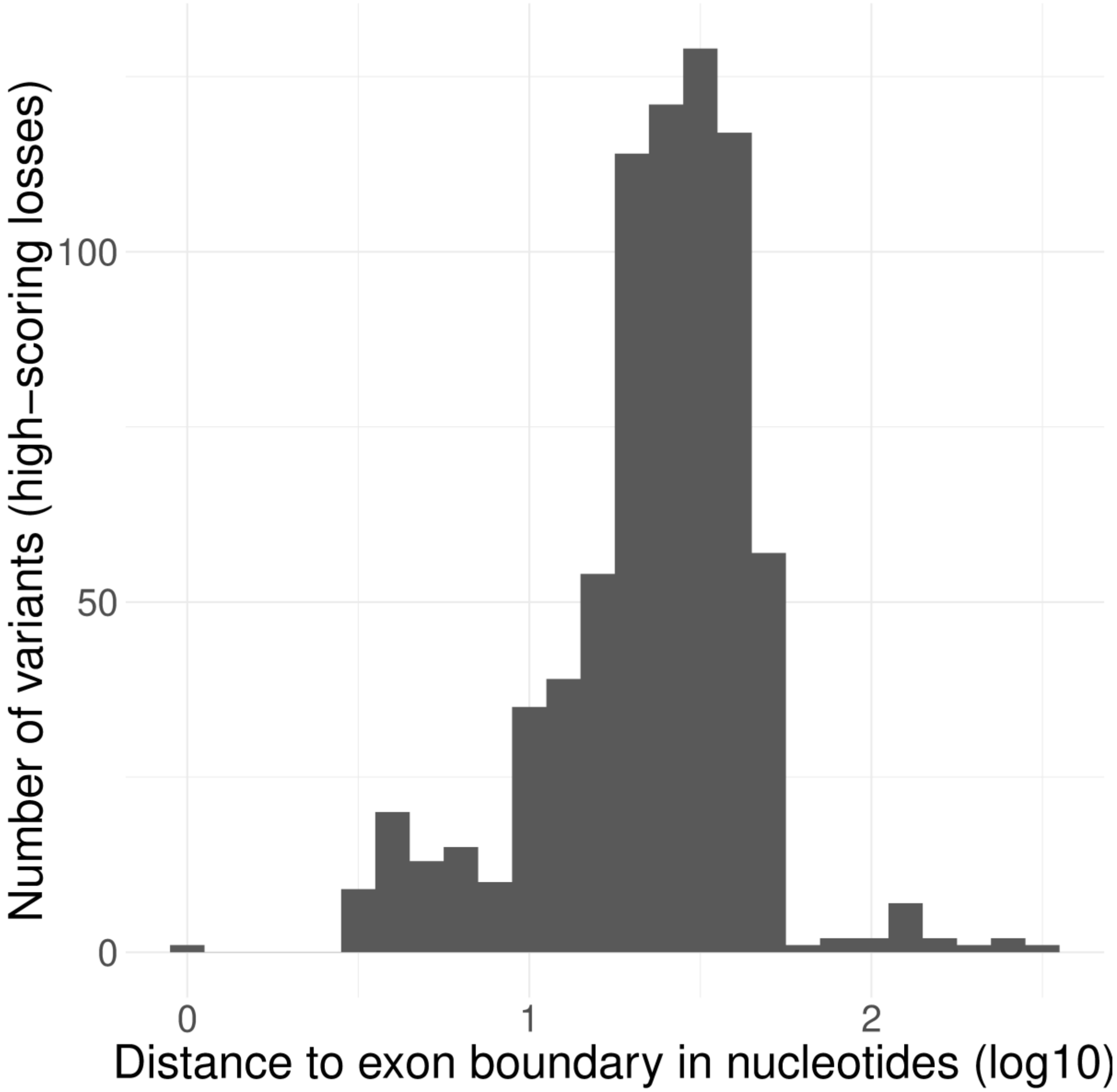

Figure S9. Distance to nearest exon junction boundary in high-scoring synonymous splice-site losses. Values are shown on logarithmic scale.

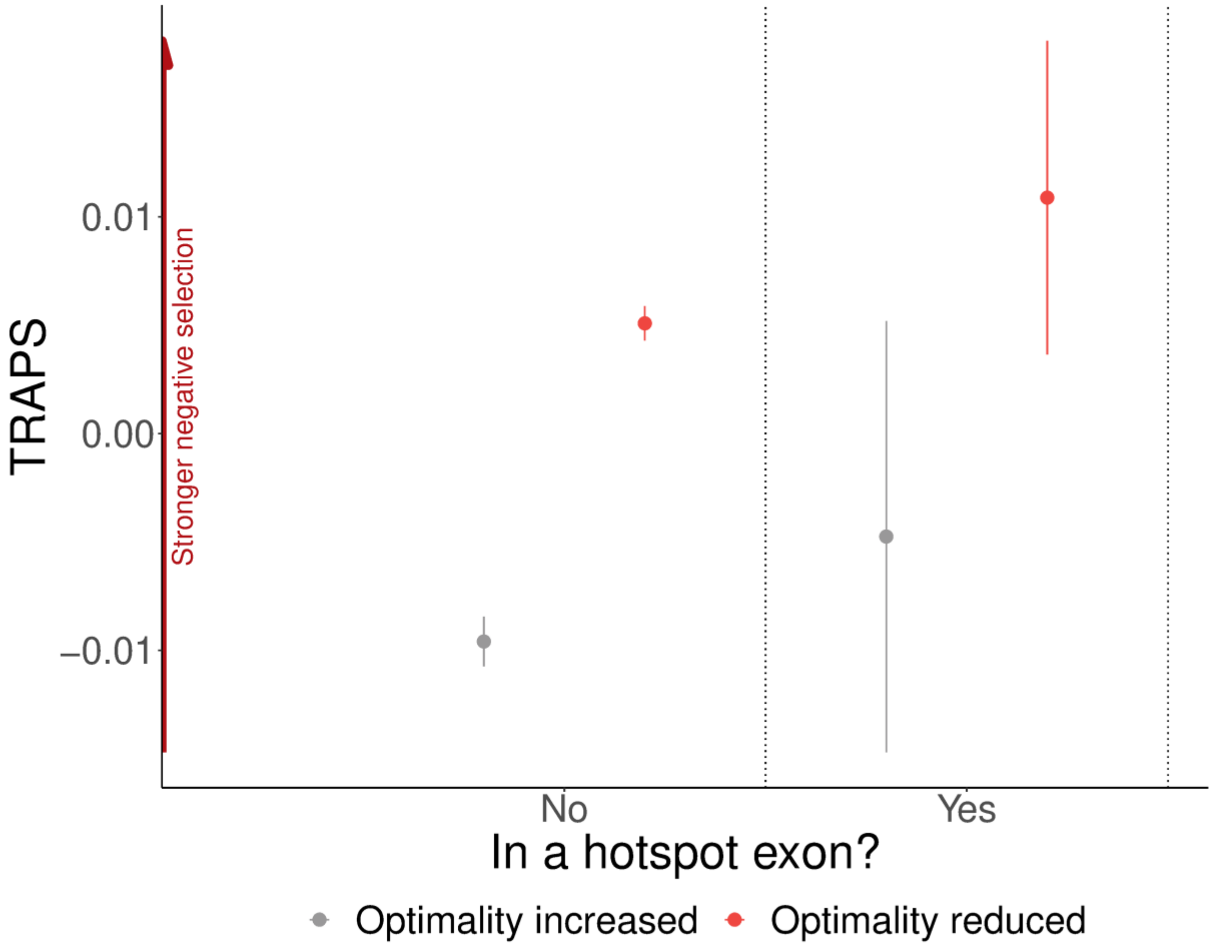

Figure S10. TRAPS scores for optimality-reducing and optimality-increasing synonymous SNVs within and outside of splicing hotspot exons.

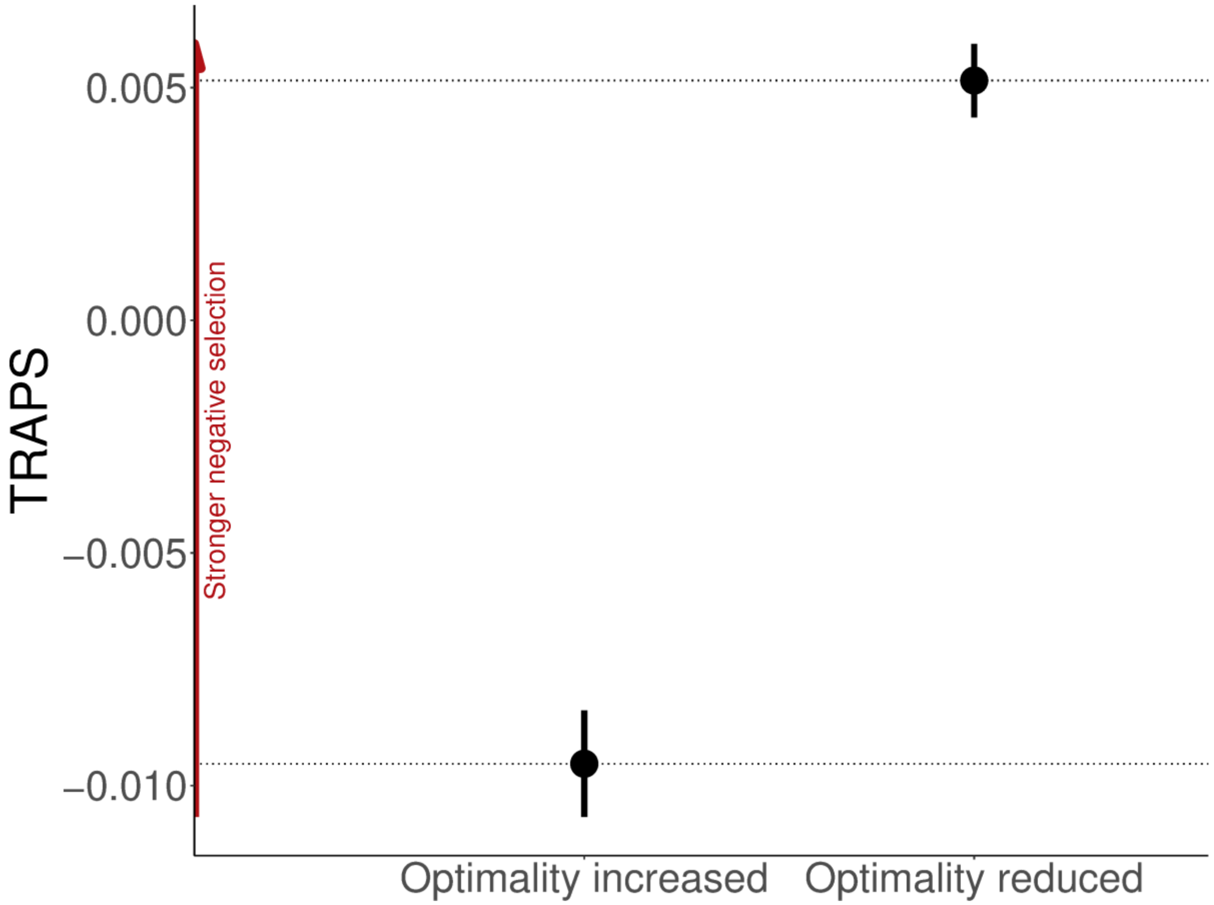

Figure S11. Averaged TRAPS scores of all optimality-reducing and optimality-increasing synonymous SNVs. Error bars are 95% binomial confidence intervals.

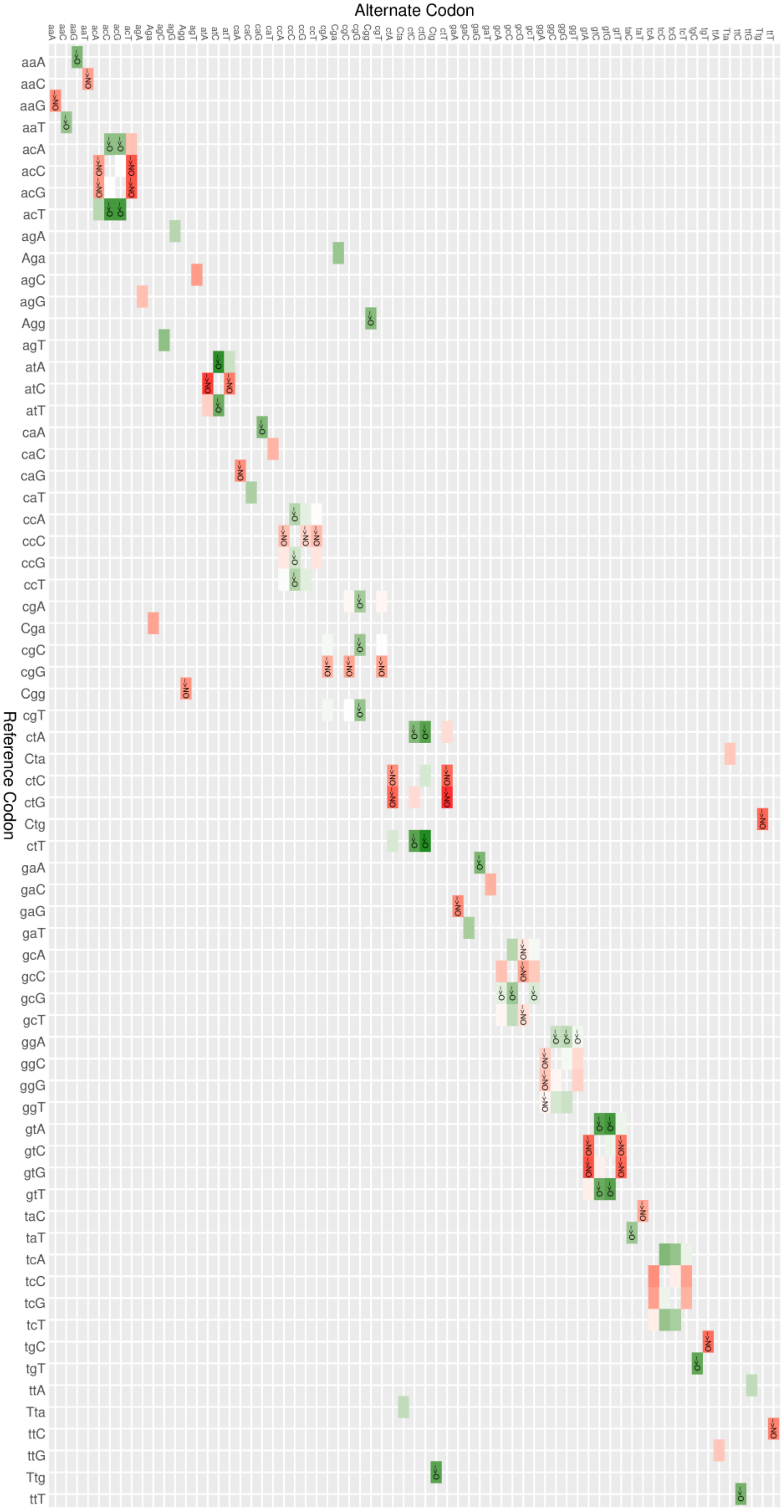
Alternate Codon
Reference Codon
aaA
aaC
aaG
aaT
acA
acC
acG
acT
agA
Aga
agC
agG
Agg
agT
atA
atC
atT
caA
caC
caG
caT
ccA
ccC
ccG
ccT
cgA
Cga
cgC
cgG
Cgg
cgT
ctA
Cta
ctC
ctG
Ctg
ctT
gaA
gaC
gaG
gaT
gcA
gcC
gcG
gcT
ggA
ggC
ggG
ggT
gtA
gtC
gtG
gtT
taC
taT
tcA
tcC
tcG
tcT
tgC
tgT
ttA
Tta
ttC
ttG
Ttg
ttT

Figure S12. Yield in optimality for all codon transitions based on CSC in HEK293T cells. Transitions that produce a more optimal codon are coloured green, whereas optimality-reducing transitions are coloured red. When the produced and alternate codons have different signs, such transition is marked with either “->O” (optimality-increasing) or “->NO” (optimality-reducing).

Supplemental Tables

Table S1. Difference in GERP distributions between optimality-reducing and optimality-increasing synonymous variants in two-codon amino acids. Bonferroni-adjusted p-values shown are from one-sided Mann-Whitney-Wilcoxon test. All genes.

Amino acid	Difference in medians	Wilcoxon p-value
Q	3.6	<0.0001
Y	1.9	<0.0001
H	1.8	<0.0001
K	1.4	<0.0001
N	1.3	<0.0001
E	1.1	<0.0001
F	0.92	<0.0001
D	0.88	<0.0001
C	0.51	<0.0001

Table S2. Predictors of the negative selection captured by TRAPS.

Predictor	Variance explained (%)	Selected by LASSO
tAI gain	16.48	Yes
CSC gain	4.22	Yes
GC gain	0.74	Yes
Mutability	–	No
Number of codons	–	No

Supplemental Note 1: gnomAD and TRAPS

We applied several criteria in the filtering and preprocessing of gnomAD data. We only retained those synonymous variants that were compliant with quality control (non-zero allele frequency and "PASS" filter flag) and had "synonymous_variant" as their most severe transcript consequence. The latter condition removes all pLoF SNVs, including variants in canonical splice sites. We additionally checked that all those variants have a protein-coding transcript where they are annotated as synonymous.

We also applied an allele number threshold to make sure that we only include those SNVs that were called in at least 80% of potential carriers. For example, for chromosome Y variants, we excluded all variants where the call was made in less than 80% of the total of 67,961 potential

male carriers, whereas for variants in autosomes, the number was 80% of two times the total number of male and female samples (125,748).

We used the same methylation scale as in the 2020 gnomAD flagship paper with values 0, 1 and 2 to distinguish between unmethylated regions and regions with medium or high levels of methylation.

Supplemental Note 2: LASSO

We used the least absolute shrinkage and selection operator (LASSO) method to perform variable selection in a linear model with negative selection (TRAPS) scores as the predictor variable and several potential predictors of this selection as explanatory variables.

We first precomputed the optimal value for the regularisation parameter λ minimising the mean-squared error using 10-fold cross-validation. Using the obtained value, we then applied LASSO, which removed such variables as the total number of codons and mutability, while keeping tAI, CSC and GC gain variables (Table S2).
